# Supplementary material for: Ctf4 Is a Hub in the Eukaryotic Replisome that Links Multiple CIP-Box Proteins to the CMG Helicase
Source: Mol Cell. 2016 Aug 4;63(3):385–96. doi: 10.1016/j.molcel.2016.06.009 (PMC4980431; doi:10.1016/j.molcel.2016.06.009)
Supplement: Document S1. Figures S1–S5 and Table S1 [file mmc1.pdf]

Molecular Cell, Volume *63*

Supplemental Information

Ctf4 Is a Hub in the Eukaryotic Replisome that Links Multiple CIP-Box Proteins to the CMG Helicase

Fabrizio Villa, Aline C. Simon, Maria Angeles Ortiz Bazan, Mairi L. Kilkenny, David Wirthensohn, Mel Wightman, Dijana Matak-Vinkovíc, Luca Pellegrini, and Karim Labib

Villa et al Supplementary Figure 1

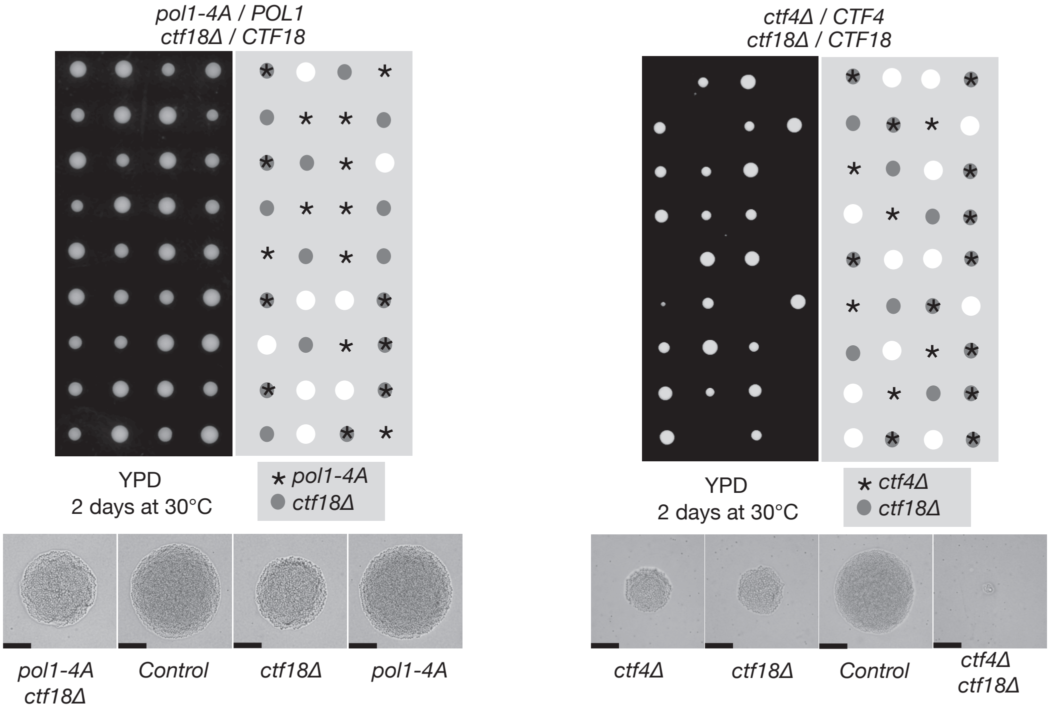

Villa et al Supplementary Figure 2

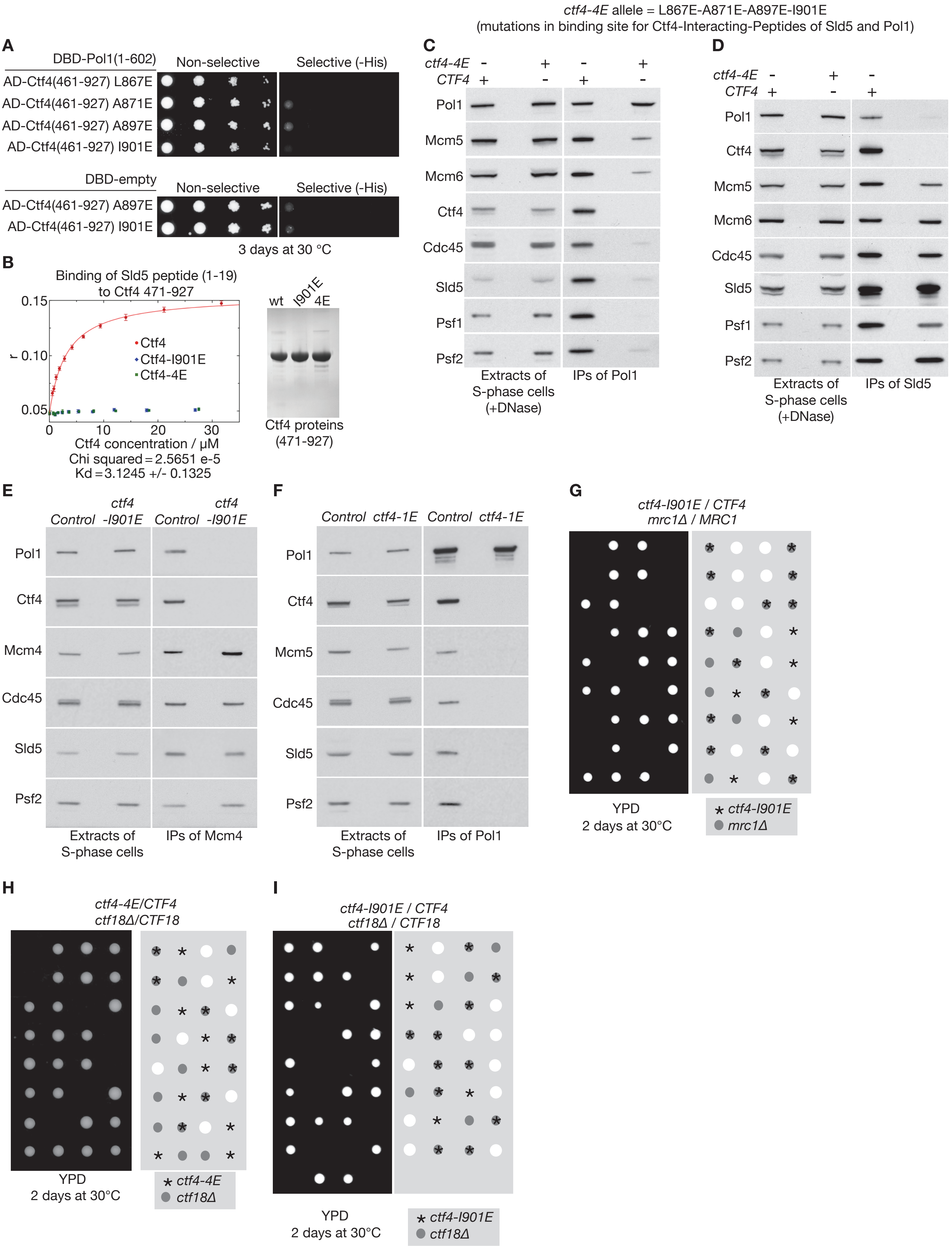

Villa et al Supplementary Figure 3

A

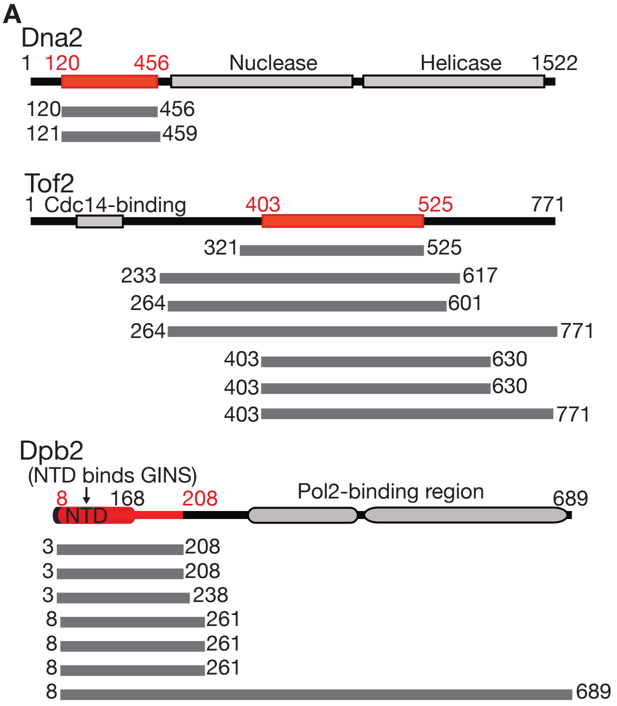

B

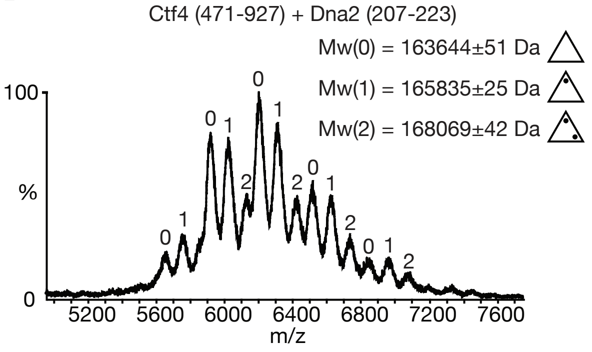

C

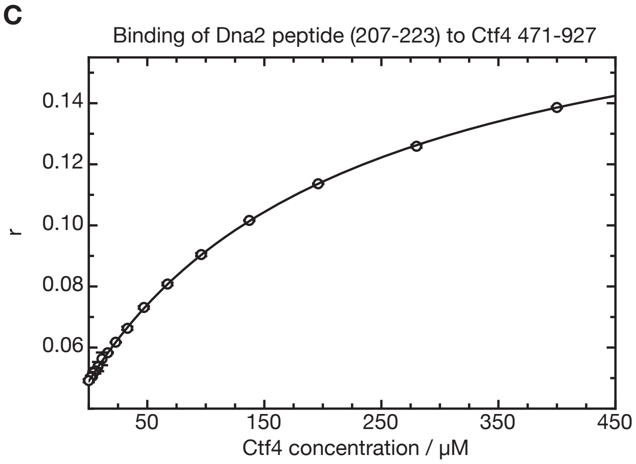

Chi squared = 5.1970
Goodness of fit = 0.9903

Parameters: Standard deviations:
Kd = 229.0088 Kd = 12.7404

Kd 95% Confidence intervals 205.6937 ... 256.0578
(based on 500 converging iterations)

D

14 Budding yeast proteins with peptides matching the pattern:
[ND]-X-D-D-I-L-X-X-J,
where [ND] = N or D, J = hydrophobic residue and
X = any residue

Sld5 4-NIDDILAEL-12
(subunit of GINS component of CMG DNA helicase)

Dna2 210-NIDDILDDI-218
(Nuclease with role in DNA replication and recombination)

Pol1 139-NFDDILGEF-147
(Catalytic subunit of DNA polymerase alpha)

Chl1 701-DGDDILSGY-709
(DNA helicase important for establishment of sister chromatid cohesion)

Lcd1/Ddc2 13-DDDDILLEL-21
(essential partner of Mec1 protein kinase for S-phase checkpoint response)

Isw2 681-DIDDILKKG-689
(ATPase involved in chromatin remodeling)

Irc20 734-NIDDILISM-742
(E3 ubiquitin ligase and putative helicase involved in recombination)

Rfx1 681-NLDDILEMM-689
(Transcriptional repressor of DNA-damage induced genes)

Pex19 176-NIDDILSQL-184
(chaperone and import receptor for peroxisomal membrane proteins)

Cog7 9-DDDDILSMF-17
(Protein trafficking in the Golgi)

Pdr8 204-NQDKDDILSLL-212
(Transcription factor controlling membrane transporters)

Vps45 453-DKDDILTEL-461
(Vacuolar Protein Sorting)

Tub4 115-NQDDILNKI-123
(Gamma Tubulin)

Sia1 590-NLDDILDEI-598
(Unknown function)

Villa et al Supplementary Figure 4

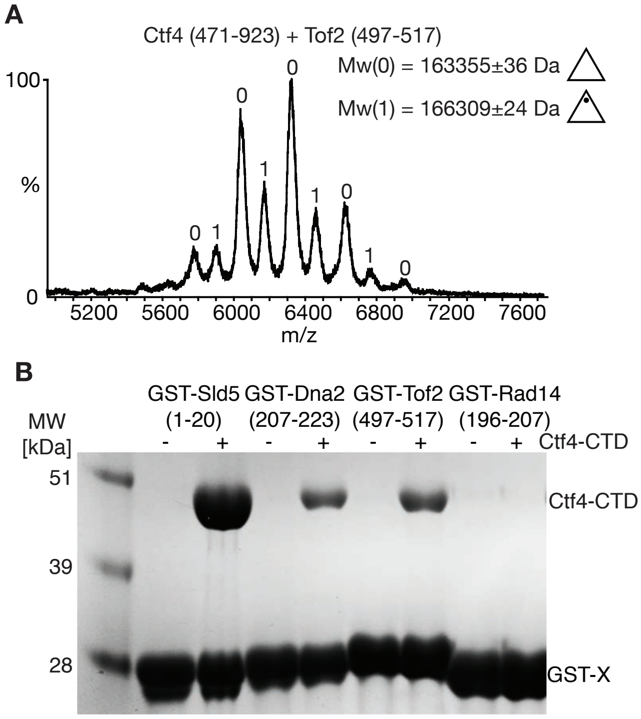

Villa et al Supplementary Figure 5

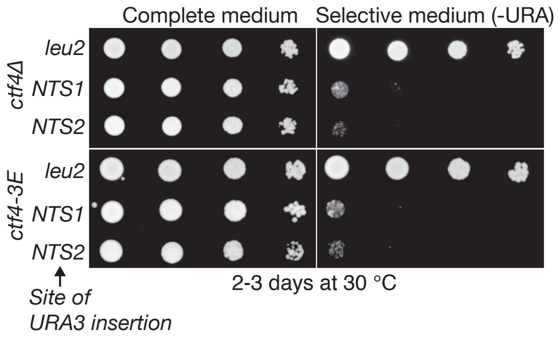

Legends to Supplemental Figures and Table

Figure S1, related to main Figure 1

pol1-4A does not share the synthetic lethal phenotype of *ctf4Δ* with *ctf18Δ*. The indicated diploids were sporulated and tetrads were dissected. The photos in the lower panels were taken after 20 hours growth at 30°C (scale bars = 50*μ*m), and genotypes were determined by replica plating after two days of growth.

Figure S2, related to main Figure 2

Additional phenotypes of mutations in the C-terminal peptide-binding domain of Ctf4. (**A**) Single mutations in the C-terminal peptide-binding domain of Ctf4 block interaction with Pol1 amino terminus in the yeast two-hybrid assay. (**B**) Fluorescence anisotropy showing that the Ctf4-I901E and Ctf4-4E mutations blocked binding to a peptide containing the Sld5 CIP-box. (**C**) Extracts of *ctf4-4E POL1-6HA* (YFV12) and *POL1-6HA* control cells (YMP262-1) were grown as in Figure 2D. Pol1-6HA was isolated from cell extracts by immunoprecipitation on anti-HA beads, and the associated proteins monitored by immunoblotting. (**D**) A similar experiment was performed with *ctf4-4E TAP-SLD5* (YFV9) and *TAP-SLD5* control cells (YSS47). (**E**) Extracts of *ctf4-I901E MCM4-9MYC* (YFV33) and *MCM4-9MYC* control cells (YSS75) were grown as in Figure 2D. Mcm4-9MYC was isolated from cell extracts by immunoprecipitation on anti-MYC beads, and the associated proteins monitored by immunoblotting. (**F**) A similar experiment was performed with *ctf4-I901E POL1-6HA* (YFV32) and *POL1-6HA* control

cells (YMP262-1). (**G**) *ctf4-I901E* is synthetic lethal with *mrc1Δ*. Cells were processed as in Figure 1A. (**H**) *ctf4-4E* is synthetic lethal with *ctf18Δ*. (**I**) *ctf4-I901E* is synthetic lethal with *ctf18Δ*.

Figure S3, related to main Figure 3

A screen for novel partners of Ctf4 461-927, and characterisation of the CIP-box of Dna2. (**A**) Summary of the novel hits in the two hybrid screen against Ctf4 461-927. Each grey line indicates a single hit in the screen, and the red segments indicate the minimal fragment of each protein that is predicted to interact with Ctf4. (**B**) Native mass spectrometry was used to show that Ctf4 471-927 binds directly to the Dna2 CIP *in vitro*. (**C**) Fluorescence anisotropy confirmed binding of Ctf4 471-927 with a Kd of 229μM. (**D**) Results of Pattern Match search of the yeast proteome (http://www.yeastgenome.org/cgi-bin/PATMATCH/nph-patmatch), using the indicated sequence that is based on the CIP-boxes of Sld5-Pol1-Dna2.

Figure S4, related to main Figure 4

The Tof2 CIP binds directly to Ctf4 471-927 *in vitro*. (**A**) Native mass spectrometry showed that Ctf4 471-927 binds directly to the Tof2 CIP-box. (**B**) GST-fusions of the indicated peptides were tested for binding to Ctf4 471-927. A peptide from the Rad14 protein provided a negative control.

Figure S5, related to main Figure 6

Transcriptional silencing in the rDNA repeats is not defective in *ctf4-3E* or *ctf4Δ* cells. Cells were processed as in Figure 6D.

Table S1, related to main figure 1

Strains used in this study - all based on the W303 yeast genetic background, except for the yeast two-hybrid strain PJ69-4A.

Strain	Genotype
W303-1	*MAT***a** *ade2-1 ura3-1 his3-11,15 trp1-1 leu2-3,112 can1-100 / MATα ade2-1 ura3-1 his3-11,15 trp1-1 leu2-3,112 can1-100*
W303-1a	*MAT***a** *ade2-1 ura3-1 his3-1 trp1-1 leu2-3, 112 can1-100*
PJ69-4A	*MAT***a** *trp1-901 leu2-3,112 ura3-52 his3-200 gal4Δ gal80Δ LYS2::GAL1-HIS3 GAL2-ADE2 met2::GAL7-la*
YAG132	*MAT***a** */ MATα CTF4 / ctf4Δ::kanMX MRC1 / mrc1Δ::hphNT*
YCE449	*MAT***a** *pol1-4A (URA3)*
YDP14	*MAT***a** */ MATα CTF4 / ctf4Δ::kanMX CTF18 / ctf18Δ::hphNT*
YFV9	*MAT***a** *ctf4-4E TAP-SLD5 (kanMX) pep4Δ::ADE2*
YFV11	*MAT***a** *ctf4-4E*
YFV12	*MAT***a** *ctf4-4E POL1-6HA (K.l. TRP1) pep4Δ::ADE2*
YFV13	*MAT***a** *ctf4-4E MCM4-9MYC (kanMX) pep4Δ::ADE2*
YFV17	*MAT***a** *dna2-4A-9MYC* (*K.l.TRP1*)
YFV21	*MAT***a** *GAL-DNA2-9MYC* (*kanMX & K.l. TRP1*) *pep4Δ::ADE2*
YFV22	*MAT***a** *GAL-DNA2-9MYC* (*kanMX & K.l. TRP1*) TAP-CTF4 (*kanMX*) *pep4Δ::ADE2*
YFV30	*MAT***a** tof2*Δ::URA3*
YFV31	*MAT***a** *ctf4-3E*
YFV33	*MAT***a** *ctf4-I901E MCM4-9MYC (kanMX) pep4Δ::ADE2*
YFV36	*MAT***a** *tof2-4A*
YFV47	*MAT***a** *ura3:: pRS306-GAL-ProteinA-CBP-TEV-TOF2 (URA3) pep4Δ::ADE2*
YFV55	*MAT***a** */ MATα ctf4-4E MRC1 / mrc1Δ::K.l.TRP1*
YFV56	*MAT***a** */ MATα ctf4-4E CTF18 / ctf18Δ::K.l.TRP1*
YFV57	*MAT***a** */ MATα ctf4-I901E CTF18 / ctf18Δ::K.l.TRP1*
YFV58	*MAT***a** */ MATα ctf4-I901E MRC1 / mrc1Δ::K.l.TRP1*
YFV59	*MAT***a** */ MATα MRC1 / mrc1Δ::K.l.TRP1 POL1 / pol1-4A (URA3)*
YFV60	*MAT***a** */ MATα CTF18 / ctf18Δ::K.l. TRP1 POL1 / pol1-4A (URA3)*
YFV62	*MAT***a** */ MATα DNA2 / dna2-4A MEC1 / mec1Δ::HIS sml1Δ::ADE2/ sml1Δ::ADE2*
YHM160	*MAT***a** *ctf4Δ::kanMX*
YMP262-1	*MAT***a** *POL1-6HA (K.l. TRP1) pep4Δ::ADE2*
YSS47	*MAT***a** *TAP-SLD5 (kanMX) pep4Δ::ADE2*
YSS75	*MAT***a** *MCM4-9MYC (kanMX) pep4Δ::ADE2*
