# Supplementary material for: Ctf4 Is a Hub in the Eukaryotic Replisome that Links Multiple CIP-Box Proteins to the CMG Helicase
Source: Mol Cell. 2016 Aug 4;63(3):385–96. doi: 10.1016/j.molcel.2016.06.009 (PMC4980431; doi:10.1016/j.molcel.2016.06.009)
Supplement: Document S2. Article plus Supplemental Information [file mmc2.pdf]

Molecular Cell

Article

Ctf4 Is a Hub in the Eukaryotic Replisome that Links Multiple CIP-Box Proteins to the CMG Helicase

Graphical Abstract

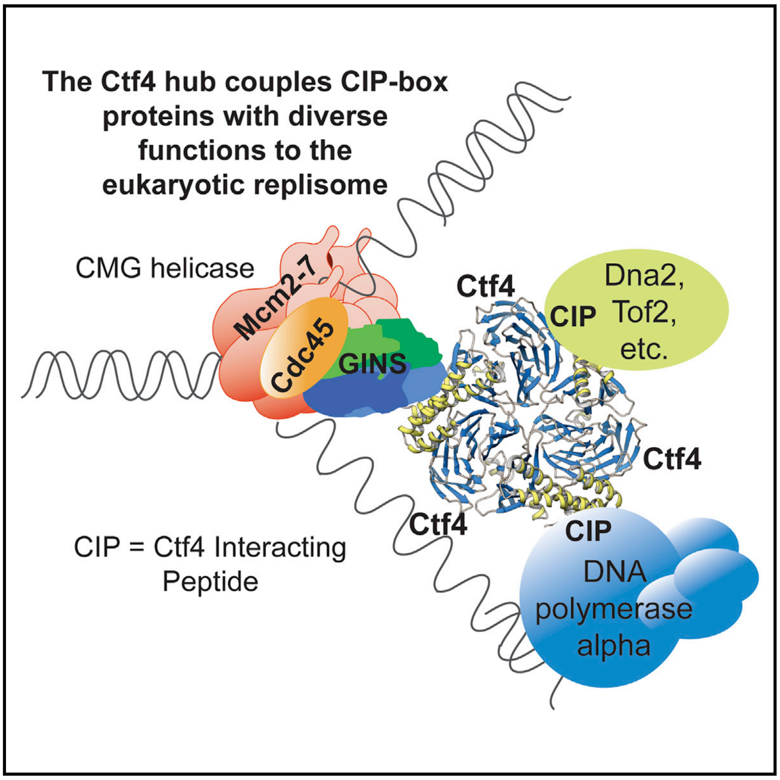

Authors

Fabrizio Villa, Aline C. Simon, Maria Angeles Ortiz Bazan, ..., Dijana Matak-Vinkovíc, Luca Pellegrini, Karim Labib

Correspondence

lp212@cam.ac.uk (L.P.), kpmlabib@dundee.ac.uk (K.L.)

In Brief

Villa et al. identify Ctf4 as a key hub in the eukaryotic replisome. Rather than simply linking DNA helicase to DNA polymerase, Ctf4 couples the replication machinery to multiple factors that contribute to diverse aspects of chromosome duplication.

Highlights

- Ctf4 is a hub that links factors with diverse functions to the eukaryotic replisome
- Multiple Ctf4 partners bind via short sequences called “CIP-boxes”
- The CIP-boxes of Dna2 and Tof2 bind to distinct sites on Ctf4
- Interaction of Dna2 and Tof2 with Ctf4 is important for rDNA copy number maintenance

Accession Numbers

5HOG
5HOI

Villa et al., 2016, Molecular Cell *63*, 385–396
August 4, 2016 © 2016 The Authors. Published by Elsevier Inc.
http://dx.doi.org/10.1016/j.molcel.2016.06.009

CrossMark

Molecular Cell
Article

CellPress

Ctf4 Is a Hub in the Eukaryotic Replisome that Links Multiple CIP-Box Proteins to the CMG Helicase

Fabrizio Villa,[1,4] Aline C. Simon,[2,4] Maria Angeles Ortiz Bazan,[1] Mairi L. Kilkenny,[2] David Wirthensohn,[3] Mel Wightman,[1] Dijana Matak-Vinkovíc,[3] Luca Pellegrini,[2,*] and Karim Labib[1,*]
[1]MRC Protein Phosphorylation and Ubiquitylation Unit, Sir James Black Centre, School of Life Sciences, University of Dundee, Dow Street, Dundee DD1 5EH, UK
[2]Department of Biochemistry, University of Cambridge, 80, Tennis Court Road, Cambridge CB2 1GA, UK
[3]Department of Chemistry, University of Cambridge, Lensfield Road, Cambridge CB2 1EW, UK
[4]Co-first author
*Correspondence: lp212@cam.ac.uk (L.P.), kpmlabib@dundee.ac.uk (K.L.)
http://dx.doi.org/10.1016/j.molcel.2016.06.009

SUMMARY

Replisome assembly at eukaryotic replication forks connects the DNA helicase to DNA polymerases and many other factors. The helicase binds the leading-strand polymerase directly, but is connected to the Pol α lagging-strand polymerase by the trimeric adaptor Ctf4. Here, we identify new Ctf4 partners in addition to Pol α and helicase, all of which contain a "Ctf4-interacting-peptide" or CIP-box. Crystallographic analysis classifies CIP-boxes into two related groups that target different sites on Ctf4. Mutations in the CIP-box motifs of the Dna2 nuclease or the rDNA-associated protein Tof2 do not perturb DNA synthesis genome-wide, but instead lead to a dramatic shortening of chromosome 12 that contains the large array of rDNA repeats. Our data reveal unexpected complexity of Ctf4 function, as a hub that connects multiple accessory factors to the replisome. Most strikingly, Ctf4-dependent recruitment of CIP-box proteins couples other processes to DNA synthesis, including rDNA copy-number regulation.

INTRODUCTION

Chromosome replication is one of the most complex processes in cell biology and is mediated by an extensive set of proteins, particularly in eukaryotes where DNA synthesis is coupled to a variety of other processes, such as chromatin regeneration, checkpoint signaling, and the establishment of cohesion between sister chromatids. Of the many factors that mediate chromosome duplication, a core assembles around the essential DNA helicase at replication forks to form a dynamic assembly called the replisome (Yao and O'Donnell, 2010). The reasons for replisome assembly are understood poorly in eukaryotes, where replisome structure is ill defined, multiple components are still of unknown function, and in vitro reconstitution of chromosome duplication is still at an early stage (Yeeles et al., 2015).

By comparison with eukaryotes and archaea, the structure and function of the *E. coli* replisome are very well characterized. A defining feature of the bacterial replisome is that the clamp loader connects the DnaB helicase to three copies of the DNA polymerase III complex that jointly synthesize the leading and lagging strands. The physical link between helicase and polymerases couples DNA unwinding to the rate of DNA synthesis, thus minimizing the exposure of single-strand DNA and also increasing the overall speed of fork progression (Kim et al., 1996). Although the same principles should apply to the eukaryotic replisome, the underlying molecular mechanisms are very different, as the eubacterial and eukaryotic machineries evolved separately (Georgescu et al., 2015), and the eukaryotic replisome contains many factors not found in its bacterial counterpart.

Three different DNA polymerases cooperate in the synthesis of the leading and lagging strands at eukaryotic forks (Kunkel and Burgers, 2014). Each new DNA molecule is initiated by Pol α, which synthesizes short RNA-DNA primers that are then extended by Pol ε and Pol δ to produce the leading and lagging strands. Both Pol ε and Pol α are connected to the CMG DNA helicase (CMG = Cdc45-MCM-GINS) as part of the eukaryotic replisome (Gambus et al., 2009; Langston et al., 2014; Sengupta et al., 2013; Tanaka et al., 2009). Whereas direct binding of Pol ε to CMG has been shown in vitro to couple DNA unwinding to the rate of leading-strand synthesis and is important for the rate of fork progression (Georgescu et al., 2014), Pol α is tethered indirectly to CMG (Gambus et al., 2009; Tanaka et al., 2009) by a factor known in budding yeast as Ctf4 (chromosome transmission fidelity = Ctf, referring to the screen in which the *CTF4* gene was first identified).

Ctf4 forms a homotrimer that has the potential to connect the CMG helicase to one or two Pol α complexes, via the α-helical bundle at the carboxyl terminus of each Ctf4 protomer, which binds to a short conserved motif in the GINS component of CMG and the Pol1 catalytic subunit of Pol α (Simon et al., 2014). These observations formed the basis for a model of the eukaryotic replisome, in which the CMG helicase is connected directly to the leading strand polymerase ε and indirectly by Ctf4 to two copies of lagging strand polymerase α, in order to promote efficient DNA synthesis. Here, we show

This is an open access article under the CC BY license (http://creativecommons.org/licenses/by/4.0/).

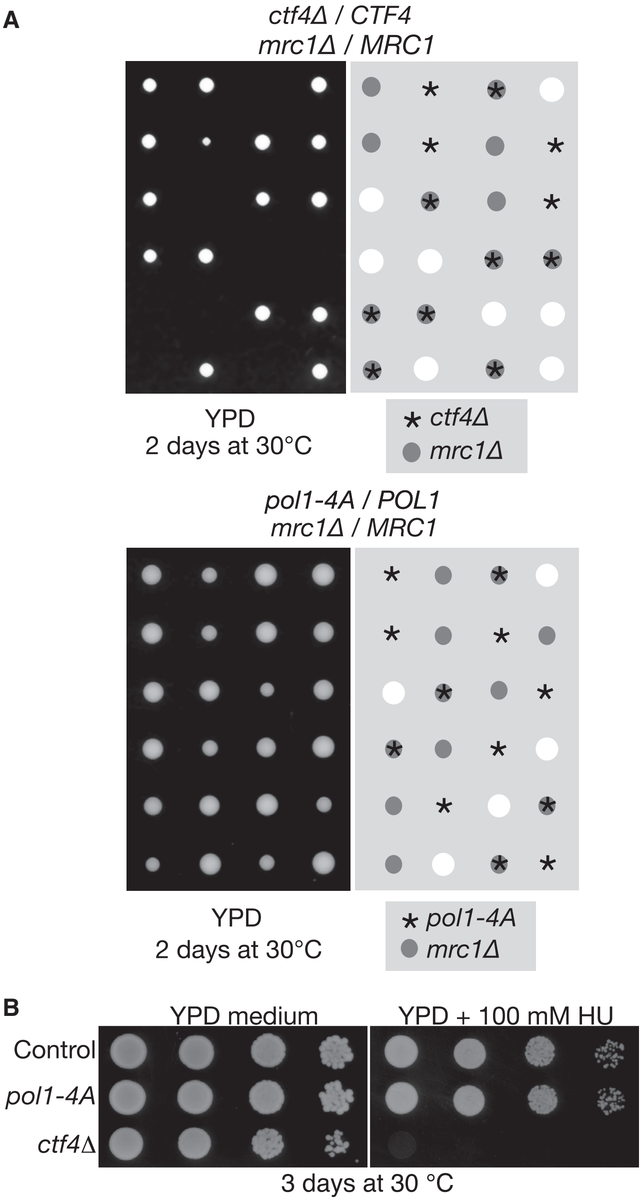

Figure 1. Displacing Pol α from the Replisome Does Not Reproduce All the Phenotypes of Deleting the *CTF4* Gene

(A) *pol1-4A* is not synthetic lethal with *mrc1*Δ. The indicated diploids were sporulated and the tetrads were dissected. The genotypes were determined by replica plating after 2 days of growth at 30°C.

(B) The *pol1-4A* allele does not cause sensitivity to hydroxyurea treatment, in contrast to *ctf4*Δ.

See also Figure S1 and Table S1.

that Ctf4 is not simply an adaptor that bridges helicase and Pol α, but instead is a nexus within the eukaryotic replisome that links multiple proteins to CMG. Our findings highlight the functional complexity of the eukaryotic replisome, in which the Ctf4 hub couples the helicase to a wide range of factors that play diverse roles in the complex process of chromosome duplication.

RESULTS

Pol α Cannot Be the Only Factor that Is Linked to the CMG Helicase by the Ctf4 C-Terminal Domain

We previously showed that mutations in the Ctf4-interacting motif of Pol1 displace Pol α from the replisome in budding yeast (Simon et al., 2014), equivalent to cells that lack Ctf4 completely (Gambus et al., 2009), suggesting that Ctf4 functions primarily as an adaptor between Pol α and the CMG helicase. However, *pol1-4A* cells lack many of the phenotypes of *ctf4*Δ cells, such as synthetic lethality with deletion of the genes encoding the checkpoint mediator Mrc1 (Figure 1A; Table 1), or the clamp loader Ctf18 (Figure S1), or sensitivity to dNTP depletion by hydroxyurea treatment (Figure 1B). These findings raised the possibility that Ctf4 might also recruit other factors to the CMG helicase, in addition to Pol α.

To explore this possibility further, we disrupted the binding site for the Ctf4-binding motifs of Pol1 and Sld5 by glutamate mutation of four exposed hydrophobic residues, L867, A871, A897, and I901, in the α-helical domain of Ctf4 (Figure 2A). As predicted, the mutated Ctf4 proteins did not interact with the amino terminus of Pol1 in the yeast two-hybrid assay (Figures 2B and S2A), but still associated with wild-type Ctf4 (Figure 2B). Moreover, the Ctf4-4E protein was able to form a trimer in vitro (Figure 2C), though neither Ctf4-4E nor Ctf4-I901E could bind in a fluorescence anisotropy assay to an isolated peptide containing the Sld5 CIP-box (Figure S2B). Upon introduction of the *ctf4-4E* or *ctf4-I901E* alleles into the native *CTF4* locus in yeast cells, the mutated proteins were expressed to a similar level as wild-type Ctf4 protein, but were unable to interact with the CMG helicase as part of the replisome, leading to greatly diminished replisome association of Pol α (Figures 2D and S2C–S2F), equivalent to cells lacking Ctf4 (Gambus et al., 2009). Crucially, both *ctf4-4E* and *ctf4-I901E* were synthetic lethal with *mrc1*Δ (Figures 2E and S2G) and *ctf18*Δ (Figures S2H and S2I). Taken together, these findings suggested that the C-terminal domain of Ctf4 might also have other client proteins in addition to Pol α.

The "CIP-Box" Motif of the Dna2 Nuclease Is Important for rDNA Maintenance

To identify novel binding partners of Ctf4, we used a yeast two-hybrid screen against residues 461–927 ($Ctf4_{CTD}$), which bridge helicase to polymerase and mediate trimerisation (Simon et al., 2014). In addition to multiple fragments from the amino terminus of Pol1 (Gambus et al., 2009), the central β-propeller domain of Ctf4 that mediates trimer formation (Simon et al., 2014), and the Psf2 subunit of GINS, we also identified three new partners of Ctf4 (Figure S3A).

The first of these was the multi-functional nuclease/helicase Dna2, which plays a role in the processing of Okazaki fragments during lagging strand synthesis, and in DNA-end resection for homologous recombination (Cejka, 2015; Kao and Bambara, 2003). We confirmed that Dna2 co-purified with Ctf4, from extracts of S phase cells in which Ctf4 forms part of the replisome (Figure 3A). Inspection of the amino acid sequence of Dna2 revealed a single peptide in its N-terminal region that closely resembled the Ctf4-interacting motif of Sld5 and Pol1 (Figure 3B). This sequence is located within the minimal fragment of Dna2

Table 1. Data Collection and Refinement Statistics for Crystallography Experiments

	Dna2 Soak	Tof2 Soak
Data Collection[a]		
Wavelength (Å)	0.91915	0.97949
Resolution (Å)	48.98–3.09 (3.23–3.09)	48.99–3.30 (3.50–3.30)
Space group	P 2 2_1 2_1	P 2 2_1 2_1
Unit cell (Å)	88.68, 99.55, and 218.37	88.58, 99.55, and 218.65
Total reflections	240,747 (28,219)	137,485 (21,087)
Unique reflections	36,111 (4,264)	29,749 (4,686)
Multiplicity	6.7 (6.6)	4.6 (4.5)
Completeness (%)	99.7 (97.7)	99.3 (98.5)
Mean I/sigma(I)	9.8 (1.7)	8.2 (2.1)
Wilson B-factor	69.49	80.76
R-merge	0.181 (1.143)	0.161 (0.773)
R-meas	0.196 (1.241)	0.182 (0.872)
CC1/2	0.994 (0.635)	0.991 (0.717)
Refinement		
Reflections used in refinement	36,057	29,704
Reflections used for R-free	1,816	1,471
R-work	0.1813	0.1789
R-free	0.2275	0.2240
Number of non-hydrogen atoms	9,598	9,650
Macromolecules	9,527	9,599
Protein residues	1,180	1,185
RMS (bonds)	0.002	0.002
RMS (angles)	0.51	0.46
Ramachandran favored (%)	95	95
Ramachandran allowed (%)	4.4	4.2
Ramachandran outliers (%)	0.26	0.43
Rotamer outliers (%)	0.095	0.19
Clashscore	3.18	3.83
Average B-factor	70.91	79.61
Macromolecules	71.05	79.75
Solvent	51.79	53.38

[a]Statistics for the highest-resolution shell are shown in parentheses.

that interacted with Ctf4 in the two-hybrid screen, and associates directly with Ctf4 in vitro when fused to glutathione S-transferase (GST), in a manner that is dependent upon residues conserved with the equivalent motifs of Sld5 and Pol1 (Figure 3B). These findings indicated that Dna2, Sld5, and Pol1 each contain a ''Ctf4-Interacting Peptide'', henceforth referred to as a CIP-box by analogy with the previously described ''PCNA-Interacting Peptide'' or PIP-box (Warbrick, 1998).

To test the importance of the CIP-box sequence for the interaction of Dna2 with Ctf4, we mutated conserved residues within the motif (Figure 3C, Dna2-4A). These mutations blocked interaction with trimeric Ctf4, both in the context of full-length Dna2 and also in the minimal Ctf4-interacting fragment identified in the two-hybrid screen (Figure 3C). We then used non-dissociative (native) mass spectrometry (Figure S3B) and fluorescence anisotropy (Figure S3C) to show that an isolated peptide containing the Dna2 CIP-box was able to bind directly to Ctf4 in vitro. Encouraged by these findings, we soaked the Dna2 peptide into crystals of Ctf4 471–927, using the same approach that we described previously (Simon et al., 2014), and found that the Dna2 CIP-box sequence folded as a two-turn α helix that bound to the helical domain of Ctf4 (Figure 3D), in a very similar manner to the CIP-boxes of Sld5 and Pol1 (Figure 3E). These data indicate that budding yeast Dna2, Sld5, and Pol1 are archetypes of a set of CIP-box proteins that all share a common mode of interaction with Ctf4.

To explore the functional significance of tethering Dna2 to Ctf4, we introduced CIP-box mutations into the endogenous *DNA2* locus in yeast cells. The *dna2-4A* allele was viable, even in the absence of the Mec1 checkpoint kinase (Figure 3F), indicating that displacement of Dna2 from Ctf4 does not produce significant defects in DNA synthesis. Interestingly, however, pulse field gel electrophoresis indicated that chromosome 12 was dramatically smaller in *dna2-4A* cells (Figure 3G), whereas other chromosomes were not affected. This suggests that tethering of Dna2 to Ctf4 at replication forks is part of a replication-coupled mechanism to maintain the large array of rDNA repeats on chromosome 12.

Tof2 and Dpb2 Define a Second Class of CIP-Box Proteins with a Distinct Binding Site in Ctf4

Multiple hits of two more new partners of Ctf4 461–927 were also identified in the screen, namely the Dpb2 subunit of Pol ε (Araki et al., 1991) and the rDNA-associated protein Tof2 (Huang et al., 2006; Park and Sternglanz, 1999). In both cases, inspection of the minimal Ctf4-interacting fragment from the two-hybrid screen identified a peptide with limited similarity to the CIP-boxes of Dna2, Sld5, and Pol1 (Figure 4A), with predicted α-helical character. Mutation of conserved residues in the putative CIP-box of Dpb2, located within the amino-terminal domain of Dpb2 that was previously shown to link Pol ε to the GINS component of the CMG helicase (Sengupta et al., 2013), abrogated interaction with Ctf4 without affecting the association of Dpb2 with GINS or the Pol2 catalytic subunit of Pol ε (Figure 4B). Similarly, mutations in the predicted CIP-box of Tof2 also abolished interaction with Ctf4 in the two-hybrid assay (Figure 4C). These findings indicated that Dpb2 and Tof2 represent two additional CIP-box proteins.

As Tof2 had not previously been shown to interact with components of the chromosome replication machinery, we expressed a tagged form of Tof2 in budding yeast cells and found that Tof2 co-purified with Ctf4 in both G1-phase and S phase (Figure 4D). This is consistent with direct association of Tof2 with Ctf4, and we confirmed by native mass spectrometry and pull-down assays that a peptide containing the Tof2 CIP-box could indeed bind directly to Ctf4 in vitro (Figures S4A and S4B). In addition, Tof2 co-purified with the CMG helicase during S phase (Figure 4D), indicating that Tof2 can associate with Ctf4 in the context of the replisome.

To establish how the divergent Tof2 CIP-box binds to Ctf4, we soaked the corresponding peptide into $Ctf4_{CTD}$ crystals.

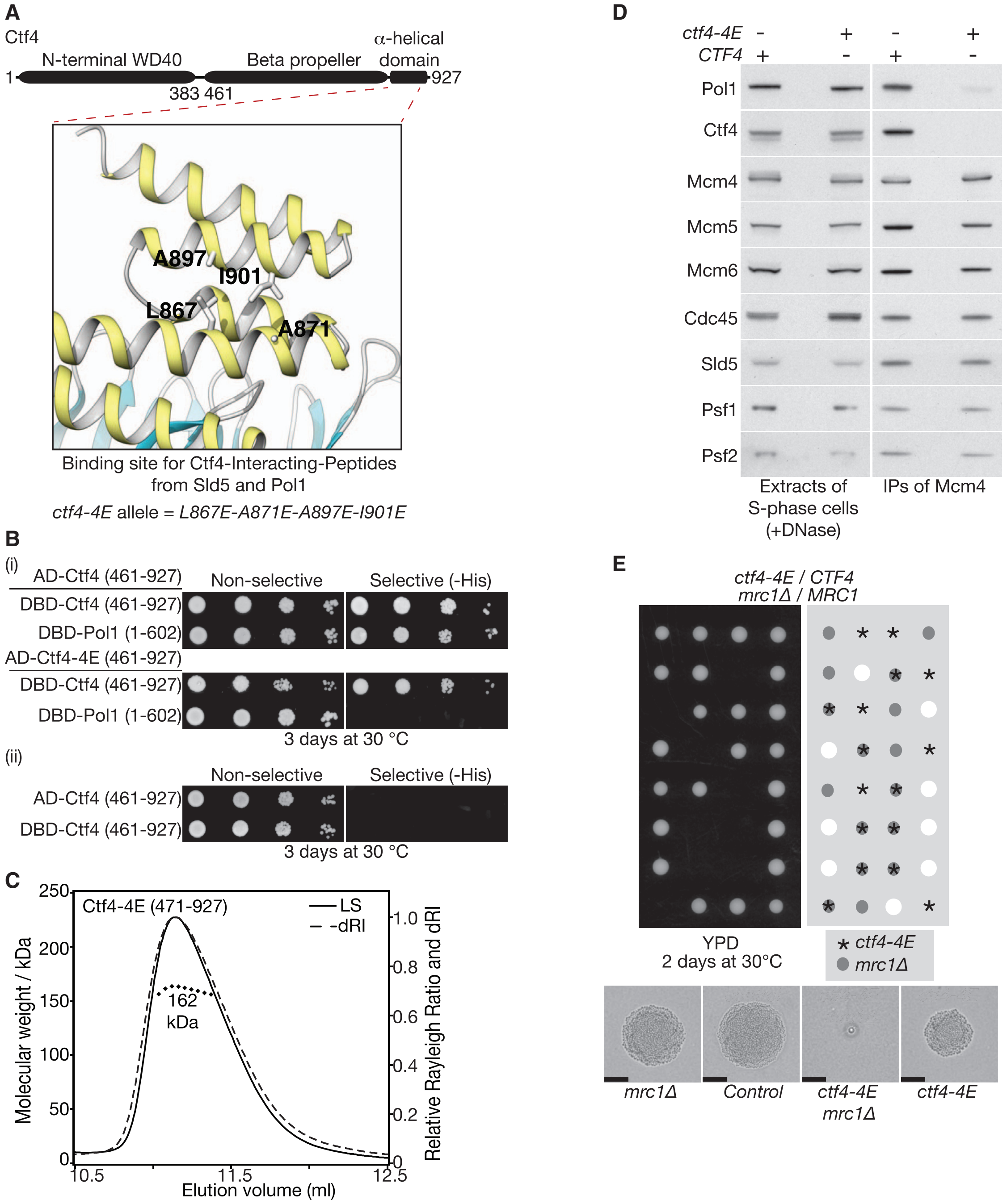

Figure 2. Mutation of the C-Terminal Peptide Binding Domain of Ctf4 Produces Many of the Phenotypes of *ctf4Δ* Cells, Indicating that This Domain has Other Partners in Addition to Pol α

(A) Illustration of the key residues in the helical domain of Ctf4 that bind to the Ctf4 interacting motifs of Sld5 and Pol1 and that are mutated in the *ctf4-4E* allele.

(B) The Ctf4-4E protein does not interact with the amino terminus of Pol1 in the yeast two-hybrid assay (AD and DBD correspond to activation and DNA-binding domains of Gal4).

(C) Size exclusion chromatography with multi-angle light scattering (SEC-MALS) indicates that Ctf4-4E 471–927 is trimeric, like wild-type Ctf4 471–927.

(D) Cultures of *ctf4-4E MCM4-9MYC* (YFV13) and *MCM4-9MYC* control cells (YSS75) were synchronized in G1-phase at 30°C and then released into S phase for 20 min. The Mcm4-9MYC was isolated from cell extracts by immunoprecipitation and the associated proteins monitored by immunoblotting.

(E) *ctf4-4E* is synthetic lethal with *mrc1Δ*. The cells were processed as in Figure 1A, and the photos in the lower images were taken after 20 hr growth at 30°C (the scale bars represent 50 μm).

See also Figure S2.

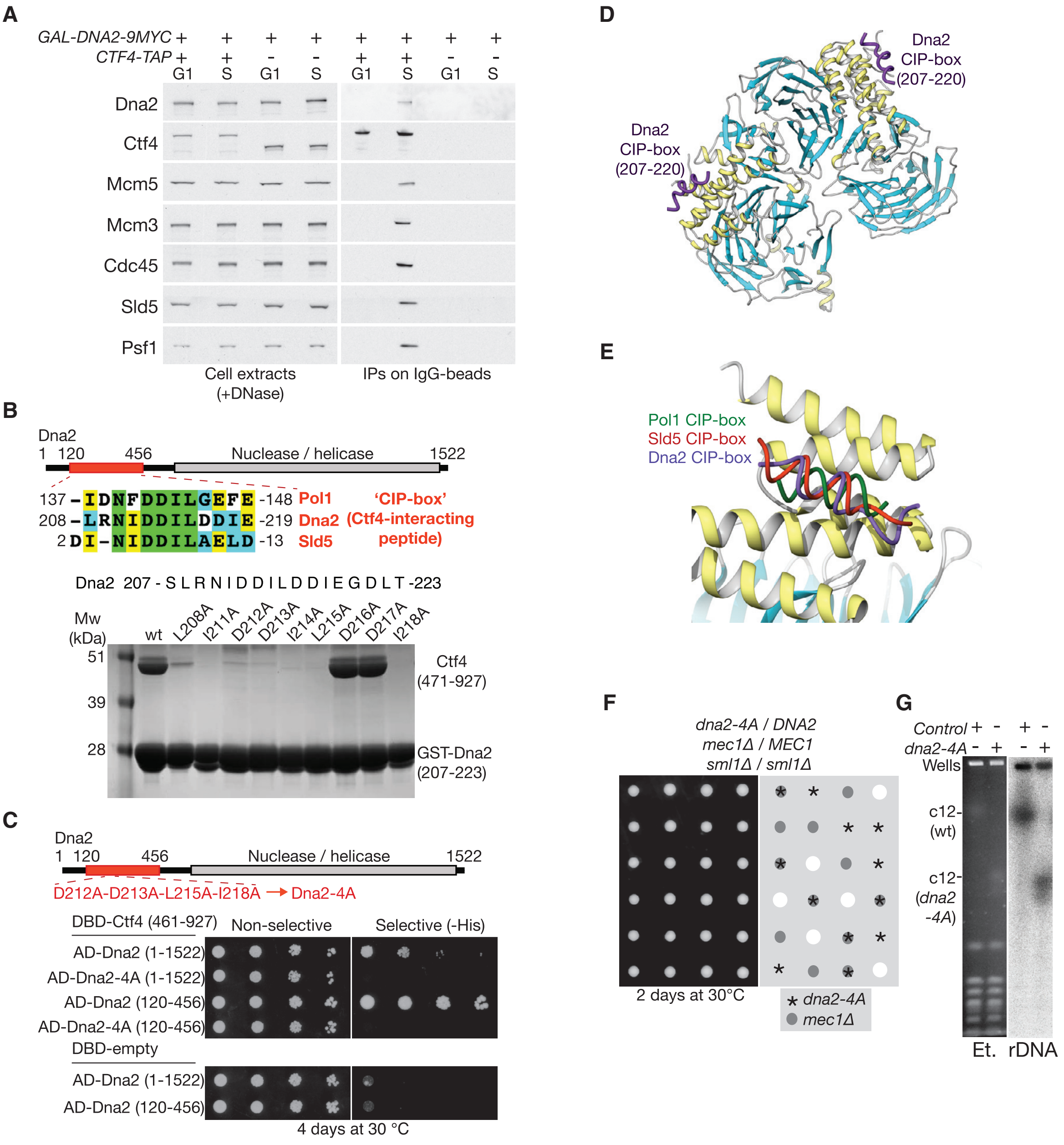

Figure 3. Interaction of the Type I CIP-Box Protein Dna2 with Ctf4 Is Important for rDNA Maintenance

(A) *GAL-DNA2-9MYC CTF4-TAP* (YFV22) and *GAL-DNA2-9MYC* control cells (YFV21) were grown at 30°C in rich medium containing 2% galactose and then treated as in Figure 2D. The Ctf4-TAP was isolated from cell extracts on magnetic beads coupled to IgG.

(B) The smallest fragment of Dna2 that interacted with Ctf4 461–927 in the two-hybrid screen (120–456 shown in red) contains a Ctf4-interacting peptide or CIP-box, closely related to those of Sld5 and Pol1 (upper). When fused to GST, the Dna2 CIP-box sequence 207–223 pulled down Ctf4 471–927 in vitro, dependent upon conserved residues (lower).

(C) Mutations in the CIP-box of Dna2 abolished the interaction of full-length Dna2 (1–1,522) or Dna2 120–456 to interact with Ctf4 461–927.

(D) When soaked into crystals of Ctf4 471–927, the Dna2 CIP-box binds to the helical region of Ctf4, as previously observed for the CIP-boxes of Pol1 and Sld5 (Simon et al., 2014). The Ctf4 protein is drawn as a ribbon, colored according to secondary structure (alpha helices in yellow and beta strands in cyan), and the Dna2 CIP-box is shown as a thin purple tube (the residues visible in the structure are indicated).

(E) Superposition of Pol1, Sld5, and Dna2 CIP-boxes bound to Ctf4 471–927 shows a common mode of interaction with the helical domain of $Ctf4_{CTD}$.

(F) *dna2-4A* is not synthetic lethal with *mec1Δ*. A diploid of the indicated genotype (YFV62) was sporulated and the tetrads dissected on YPD medium.

(G) Pulse field gel electrophoresis of chromosomal DNA from control cells (W303-1a) and *dna2-4A* (YFV17). The gel was stained with ethidium bromide (left) and then transferred to nitrocellulose before hybridization with a probe to the rDNA (right).

See also Figure S3.

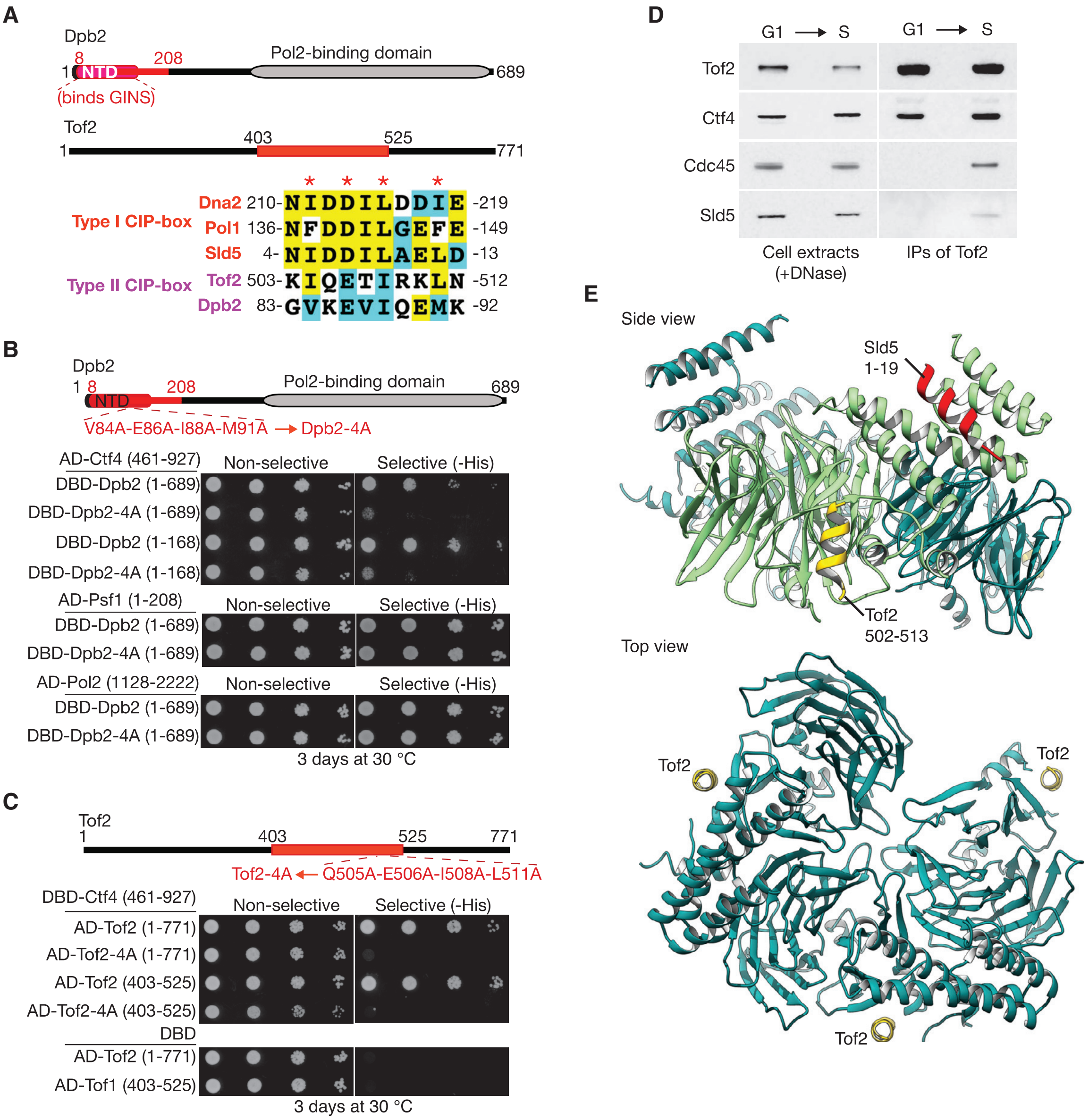

Figure 4. Tof2 and Dpb2 Define a New Class of Type II CIP-Box Proteins that Bind to a Novel Binding Pocket on the Surface of Ctf4

(A) The smallest fragments of Dpb2 and Tof2 that interacted with Ctf4 461–927 in the two-hybrid screen (shown in red) were each found to contain a putative CIP-box with limited homology to the type I CIP-boxes of Sld5, Dna2, and Pol1. These are termed type II CIP-boxes.

(B) Mutations in the type II CIP-box of Dpb2 blocked interaction in the two hybrid assay with Ctf4 461–927, without affecting interaction with the Psf1 subunit of GINS or the Pol2 catalytic subunit of Pol ε.

(C) Similarly, mutations in the type II CIP-box of Tof2 blocked interaction with Ctf4 461–927.

(D) Cells expressing *GAL-TOF2-Pro*teinA (YFV47) were grown as in Figure 3A, before isolation of Tof2-ProteinA on IgG beads. The indicated proteins were monitored by immunoblotting.

(E) Soaking of the Tof2 CIP-box peptide into crystals of Ctf4 471–927 revealed a novel binding site on the surface of Ctf4. The side view of the $Ctf4_{CTD}$ structure shows the Tof2 CIP-box peptide, in yellow (the residues visible in the structure are indicated), bound to one Ctf4 protomer, colored in lighter green to facilitate identification of the Tof2-binding site. To highlight the different binding site recognized by the type II CIP-box of Tof2, the Sld5 type I CIP-box is also shown, overlaid in red on the structure. The top view shows trimeric $Ctf4_{CTD}$ with three bound Tof2 peptides.

See also Figure S4.

Remarkably, the peptide bound to a different site to that recognized by the "type I" CIP-boxes of Dna2, Sld5, and Pol1, namely to the side of the C-terminal blade in the β-propeller domain of $Ctf4_{CTD}$ (Figure 4E). To validate these findings, we used the crystal structure to generate the *ctf4-3E* allele, by glutamate mutation of three key hydrophobic residues at the interface between Ctf4 and the Tof2 CIP-box (Figure 5A). Critically, the *ctf4-3E* mutations abolished interaction with full-length Tof2 in

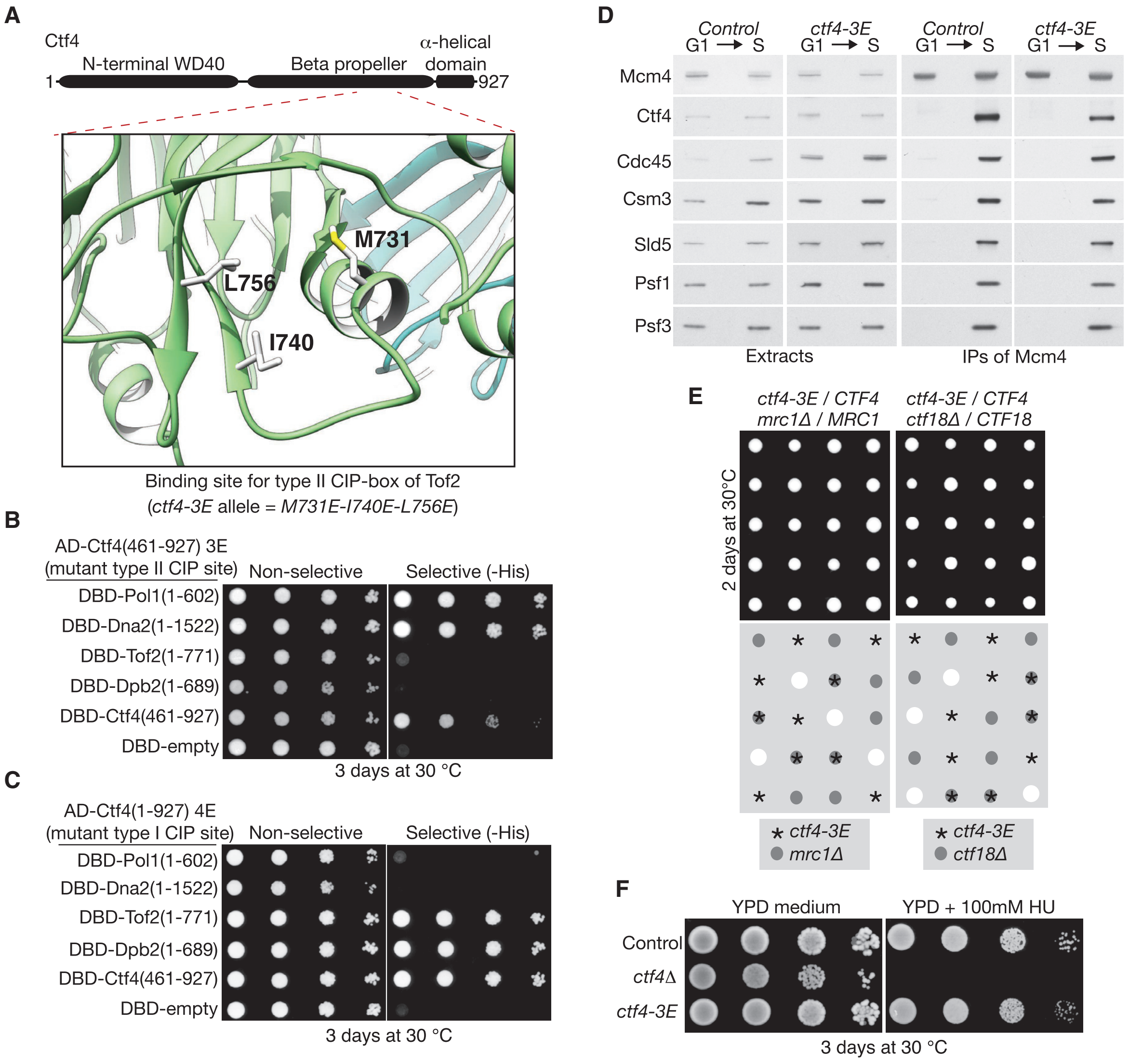

Figure 5. The *ctf4-3E* Allele Disrupts the Binding Site for Type II CIP-Box Proteins

(A) Close-up view of the Tof2 binding site on the $Ctf4_{CTD}$ surface, highlighting the position of Ctf4 amino acids M731, I740, and L756 at the binding interface, which were mutated to make the *ctf4-3E* allele.

(B) Ctf4-3E did not interact with the type II CIP-box proteins Tof2 or Dpb2 in the two-hybrid assay, though interaction with type I CIP-box proteins such as Pol1 and Dna2 was unaffected.

(C) Conversely, Ctf4-4E could not interact with type I CIP-box proteins, but still interacted with the type II CIP-box proteins Tof2 and Dpb2.

(D) Mcm4 was isolated from extracts of the indicated strains, grown as in Figure 2D.

(E) *ctf4-3E* is not synthetic lethal with *mrc1*Δ or *ctf18*Δ. The cells were processed as in Figure 2E.

(F) *ctf4-3E* cells do not share the sensitivity of *ctf4*Δ cells to growth in the presence of hydroxyurea.

the yeast two-hybrid assay, without affecting interaction with Dna2 and Pol1 (Figure 5B). Moreover, Ctf4-3E was unable to interact with Dpb2 in the same assay (Figure 5B). Conversely, Ctf4-4E (with mutated binding site for type I CIP-boxes) was still able to interact with both Tof2 and Dpb2 (Figure 5C), despite being unable to interact with Dna2 and Pol1 as described above. These findings indicate that both Tof2 and Dpb2 are archetypal "type II CIP-box proteins", with a distinct binding site on Ctf4 to the type I CIP-boxes of factors such as Dna2, Pol1, and Sld5.

Tethering of Tof2 to Ctf4 Is Important for rDNA Maintenance

We introduced the *ctf4-3E* mutations into the endogenous *CTF4* locus in yeast cells and then compared the resulting phenotypes with those of *ctf4-4E* and *ctf4-I901E*. In contrast to the effects of displacing type I CIP-box proteins from Ctf4 (*ctf4-4E* and *ctf4-I901E*; Figures 2 and S2), the Ctf4-3E mutations did not prevent association of Ctf4 with the CMG helicase (Figure 5D). Moreover, *ctf4-3E* cells were not synthetic lethal with *mrc1*Δ or *ctf18*Δ (Figure 5E) and did not show sensitivity to dNTP depletion (Figure 5F).

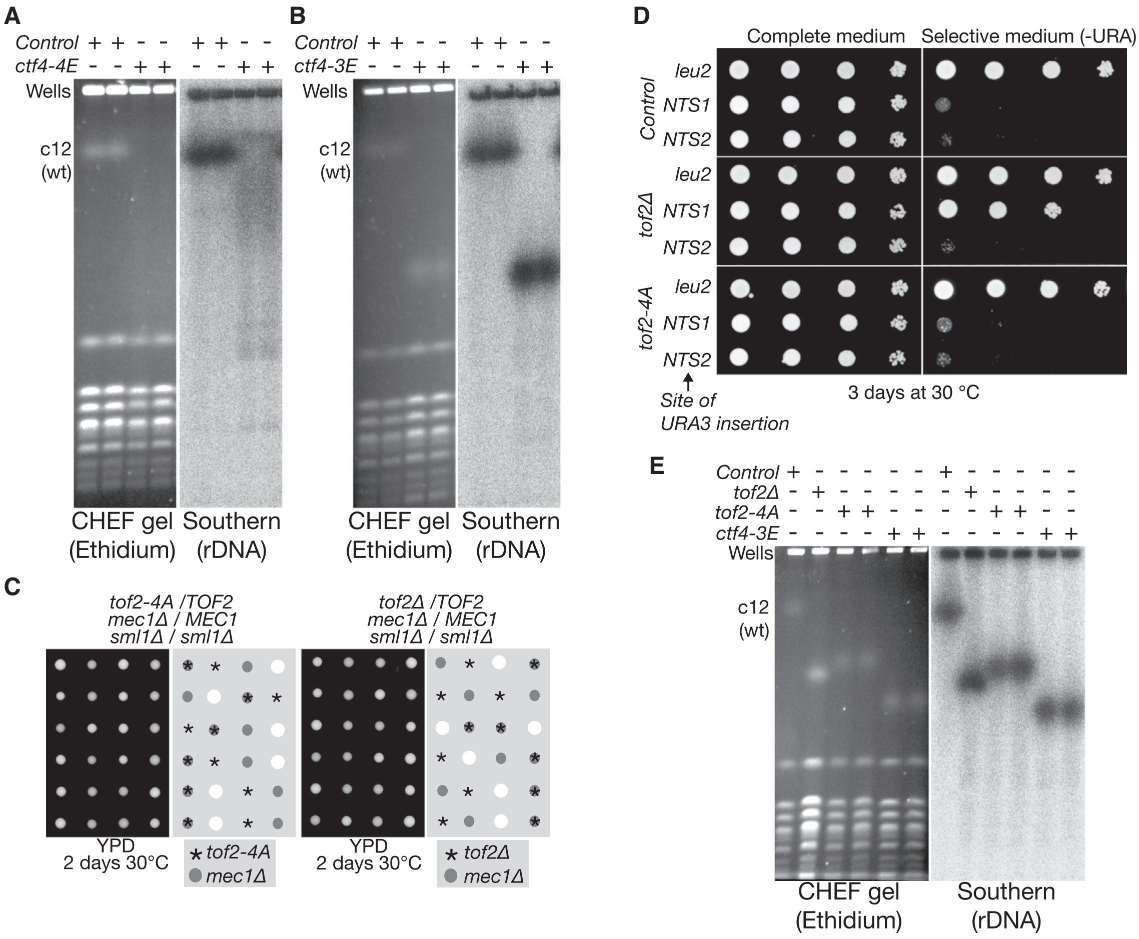

Figure 6. Interaction of the Type II CIP-Box Protein Tof2 with Ctf4 Is Important for rDNA Maintenance

(A) Pulse field gel electrophoresis of chromosomal DNA from control cells (W303-1a) and *ctf4-4E* (YFV11), as in Figure 3G.

(B) Similar experiments for control and *ctf4-3E* (YFV31).

(C) Neither *tof2-4A* nor tof2Δ is synthetic lethal with *mec1*Δ.

(D) Transcriptional silencing in the "Non-Transcribed Spacer 1" region of the rDNA repeat requires Tof2, but is independent of the association between Tof2 and Ctf4. The URA3 marker was inserted at the *leu2* locus or within the *NTS1* or *NTS2* sites within an rDNA repeat on chromosome 12. The expression of *URA3* was then monitored on selective plates for the indicated strains.

(E) The size of chromosome 12 is reduced in *tof2-4A* cells (YFV36), slightly more in *tof2*Δ (YFV30), and even more in *ctf4-3E*.

See also Figure S5.

We then used pulse field gel electrophoresis to examine maintenance of the rDNA array. The size of chromosome 12 was extremely heterogenous in *ctf4-4E* (Figure 6A) or *ctf4-I901E* (F.V. and K.L., unpublished data), indicating that the rDNA array is highly unstable when Ctf4 is unable to associate with the CMG helicase, so that all type I and type II CIP-box proteins are displaced from the replisome. In contrast, chromosome 12 was not heterogeneous in *ctf4-3E* cells compared to wild-type, but instead was much smaller (Figure 6B), indicating that the association of one or more type II CIP-box proteins with Ctf4 is important to preserve the normal size of the rDNA array.

We focused on Tof2, given its established role in rDNA biology (Corbett et al., 2010; Geil et al., 2008; Huang et al., 2006; Johzuka and Horiuchi, 2009). Neither *tof2-4A* nor *tof2*Δ were synthetic lethal with *mec1*Δ *sml1*Δ (Figure 6C), indicating that Tof2 and its association with Ctf4 are dispensable for efficient DNA synthesis at replication forks. Moreover, although Tof2 is important to preserve transcriptional silencing within the rDNA repeats (Huang et al., 2006), this did not require the association of Tof2 with Ctf4 (Figure 6D; Figure S5 shows that rDNA silencing is also not defective in either *ctf4-3E* or *ctf4*Δ). However, we found that the size of chromosome 12 was strikingly reduced in both *tof2-4A* and *tof2*Δ cells (Figure 6E), indicating that tethering of Tof2 to Ctf4 is important during chromosome replication for the preservation of rDNA copy number. Interestingly, the size of chromosome 12 is reduced still further in *ctf4-3E* cells (Figure 6E), suggesting that association of other type II CIP-box proteins with Ctf4 might also contribute to rDNA maintenance.

DISCUSSION

Although budding yeast can form colonies in the absence of Ctf4 under laboratory conditions, the cells are extremely sick, are unable to grow at low temperatures, are defective in sister chromatid cohesion, and have a very high rate of genome instability (Hanna et al., 2001; Kouprina et al., 1992; Miles and Formosa, 1992), indicating the importance of Ctf4 for efficient chromosome duplication. Similarly, fission yeast cells lacking the Ctf4 ortholog Mcl1 are very sick or unable to grow (Mamnun et al., 2006; Williams and McIntosh, 2002). In higher eukaryotes, Ctf4 has been reported to be essential for viability in *Drosophila melanogaster* and is required for replication in *Xenopus laevis* and human cells (Gosnell and Christensen, 2011; Im et al., 2009; Zhu et al., 2007). Our data indicate that these phenotypes reflect the cumulative failure to recruit multiple CIP-box proteins to the eukaryotic replisome, together with additional partners of the amino terminal WD40 domain of Ctf4 such as the E3 ligase component Mms22 in budding yeast (Buser et al., 2016; Gambus et al., 2009; Mimura et al., 2010)

In this study and our previous work, we have identified five CIP-box proteins of two different subtypes. The basic features of the CIP-box appear to be a propensity to adopt a helical conformation, plus a limited number of conserved hydrophobic and charged residues, making it likely that additional CIP-box proteins remain to be identified in future studies. For example, the list of yeast proteins with sequences that closely resemble the type I CIP-box of Sld5-Pol1-Dna2 includes the Chl1 DNA helicase (Figure S3D), and previous work showed that *chl1*Δ and *ctf4*Δ produce similar and epistatic defects in the establishment of sister chromatid cohesion (Borges et al., 2013), suggesting that they might act together. Recent work indicates that Chl1 is indeed a type I CIP-box protein that is recruited to the replisome by Ctf4, helping explain the role of the Ctf4 in cohesion establishment (Samora et al., 2016 [this issue of *Molecular Cell*]).

The way that CIP-box proteins compete with each other for binding to Ctf4 remains an interesting issue for future investigation. The pre-dominant partners of Ctf4 in extracts of wild-type yeast cells appear to be GINS (and thus the CMG helicase) and Pol α (Gambus et al., 2009), and in vitro studies of four CIP-box proteins indicate a hierarchy of affinities for Ctf4 among the isolated CIP-boxes, with the CIP-box of Sld5 binding most tightly (affinity constant K_d = 5 μM), followed by the Pol1 CIP (25 μM) (Simon et al., 2014), then Dna2 (230 μM), and finally Tof2 (K_d not determinable in our fluorescence anisotropy assay). This hierarchy (Sld5 > Pol1 > Dna2 > Tof2) is reflected in the native mass spectrometry data by the degree of occupancy by CIP-box peptides of their corresponding binding sites in Ctf4 (Figures S3B and S4A; Simon et al., 2014). Although the CIP-boxes are required for the cognate proteins to bind to Ctf4, electron microscopic studies indicate that Ctf4 has additional contacts with the CMG helicase (Simon et al., 2014), which would also contribute to the affinity. It remains possible that some of the Ctf4-client interactions are regulated within the replisome by post-translational modifications of the CIP-box proteins or of Ctf4 itself (perhaps regulating access of CIP-box motifs to Ctf4 in some cases). Alternatively, association of CIP-box proteins and Ctf4 might require additional contacts with other proteins that are only possible in the context of the replisome. Indeed, although Ctf4 binds GINS throughout the cell cycle in extracts of yeast cells (Gambus et al., 2009) and the same is true for overexpressed Tof2 (Figure 4D), the association of Ctf4 with Pol α (van Deursen et al., 2012) and Dna2 (Figure 3A) is regulated so that it is detected in S phase, but not in G1-phase.

The expanded set of CIP-box proteins leads to a revised model for the role of Ctf4 at eukaryotic replication forks. In addition to its role as a bridge between the CMG helicase and Pol α, our data indicate that Ctf4 functions as a key hub within the replisome, linking the helicase to a diverse set of partners. These findings further indicate that the function of replisome assembly in eukaryotes is not simply to ensure the efficient synthesis of two strands of DNA at replication forks, but also to couple fork progression to other processes that are important for eukaryotic chromosomes to be duplicated in all their complexity.

Notably, mutation of the CIP-box motifs of Dna2 and Tof2 does not lead to detectable defects in DNA synthesis. Instead, association of these factors with Ctf4 is particularly important to maintain the size of chromosome 12 that contains the very large array of rDNA repeats. The details remain to be explored in the future, but our findings indicate that important mechanisms for rDNA copy number regulation must be coupled to replisome function at DNA replication forks.

Whereas displacement of type II CIP-box proteins from Ctf4 leads to a reduction in the size of chromosome 12 (Figure 6B), simultaneous displacement of all type I and type II CIP-box proteins leads to a highly heterogeneous range of sizes for chromosome 12 (Figure 6A). We anticipate that further partners of Ctf4 will also contribute to rDNA copy number regulation, since chromosome 12 actually becomes larger in the complete absence of Ctf4 (Saka et al., 2016), probably reflecting the highly complex nature of replisome-coupled rDNA maintenance in budding yeast. The evolutionary conservation of these processes in other eukaryotic species will be an interesting theme to explore in future studies.

EXPERIMENTAL PROCEDURES

Yeast Methods

The strains used in this study are all based on the W303 background and are listed in Table 1. Yeast growth, two-hybrid analysis, and immunoprecipitation experiments were performed as described in detail previously (Maculins et al., 2015; Maric et al., 2014). A two-hybrid screen against amino acids 461–927 of Ctf4 was performed by the company Hybrigenics.

Sequence Analysis

Multiple sequence alignments were performed using ClustalW software and presented using Boxshade, both of which were accessed via the website Biology Workbench 3.2 (http://workbench.sdsc.edu). Secondary structure predictions were performed using the Jpred 4 server (Drozdetskiy et al., 2015).

Co-crystallization of Ctf4 471–927 with the CIP-Boxes of Dna2 and Tof2

$Ctf4_{CTD}$ crystals comprising residues 471–927 were grown as described previously (Simon et al., 2014). For co-crystallization experiments, the

peptides SLRNIDDILDDIEGDLT and SHAKDVKIQETIRKLNRFKPT, corresponding to residues 207 to 223 of yeast Dna2 and amino acids 497 to 517 of yeast Tof2, respectively, were synthesized with an amino-terminal fluorescein label (Cambridge Peptides). The Dna2 peptide was solubilized in 20 mM ammonium bicarbonate to a concentration of 630 μM; the Tof2 peptide was solubilized in 0.2 M tri-sodium citrate pH 6.2, 7.5% (w/v) PEG 8000, and 0.45 M NaCl to a concentration of 7 mM. For Dna2 peptide, soaking was performed by adding 1 μl of peptide solution to a 2 μl crystallization drop containing native $Ctf4_{CTD}$ crystals, whereas for Tof2 soaks, crystals were transferred straight into a 2 μl drop of peptide solution.

The crystals were soaked with the peptide for 24 hr at 19°C, back-soaked in crystallization buffer, and flash-frozen in liquid nitrogen. X-ray diffraction data for $Ctf4_{CTD}$ crystals soaked with the Dna2 and Tof2 peptides were collected on beamline I02 of the Diamond Light Source and processed as described previously (Simon et al., 2014). The position of the Ctf4-binding motifs of Dna2 and Tof2 in the crystals structure of $Ctf4_{CTD}$ was readily identified by inspection of F_o-F_c difference Fourier maps. Amino acids 207 to 220 of Dna2 and 502 to 513 of Tof2 were built in the electron density map and the structures of $Ctf4_{CTD}$ bound to Dna2 and Tof2 were then further refined using Coot and PHENIX Refine to R-work/R-free values of 0.181/0.227 and 0.179/0.224, respectively. MolProbity scores for the $Ctf4_{CTD}$ - Dna2 and $Ctf4_{CTD}$ - Tof2 structures were 1.43 and 1.49, respectively. Data collection and refinement statistics are given in Table 1.

Native Mass Spectrometry

In preparation for non-denaturing nano-electrospray ionization mass spectrometry (native mass spectrometry), Ctf4 471–927 was subjected to two successive rounds of buffer exchange into 500 mM ammonium acetate using illustra NAP-5 columns (GE Healthcare). Following buffer exchange, a 5-fold or 10-fold molar excess of Dna2 peptide 207- SLRNIDDILDDIEGDLT -223 or Tof2 peptide 497- SHAKDVKIQETIRKLNRFKPT -517 solubilized in 500 mM ammonium acetate was mixed with $Ctf4_{CTD}$ at a final protein concentration of 100 μM and incubated for a minimum of 30 min. Native mass spectra were recorded on a Synapt HDMS instrument (Waters) and calibrated using caesium iodide (100 mg ml^{-1}) as described previously (Hernández and Robinson, 2007; Simon et al., 2014).

Analysis of Molecular Weight of Ctf4-4E by Multi-angle Light Scattering

100 μl of $Ctf4_{CTD}$ 4E mutant protein (with mutated binding site for type I CIP-boxes) at a concentration of 2 mg/ml was loaded onto a Superdex S200 HR 10/300 gel-filtration column (GE Healthcare) in 20 mM HEPES pH 7.2, 160 mM NaCl at a flow rate of 0.5 ml/min. The column was controlled using an Äkta Purifier System (GE Healthcare) and was linked to a DAWN 8^+ 8-angle light scattering detector (Wyatt Technology) with a fused silica sample cell using a laser wavelength of 664 nm. The change in the refractive index was detected using an Optilab T-rEX refractometer with extended range (Wyatt Technology) using a wavelength of 658 nm. Data collection and analysis was carried out using the ASTRA6 software package (Wyatt Technology). Molecular weight determination across the sample peak was carried out using a Zimm-plot derived global fitting algorithm with a fit degree of 1 and a dn/dc value of 0.1850 ml/g.

GST-Pull-Downs

For each Dna2 construct to be tested for interaction with $Ctf4_{CTD}$, a 25-ml *E. coli* Rosetta2 (DE3) culture overexpressing the GST-fusion construct was pelleted, resuspended in buffer (50 mM Tris [pH 7.0], 500 mM NaCl, 10% [w/v] glycerol, 1 mM DTT, and protease inhibitors) (Sigma), and lysed by sonication. Following centrifugation, the soluble extract was mixed with 50 μl of Glutathione Sepharose beads (GE Healthcare) pre-equilibrated in the same buffer, and incubated under rotation at 4°C for 1 hr. Unbound protein was removed by three consecutive washes with 1 ml of buffer, followed by three 1-ml washes with pull-down buffer (20 mM HEPES [pH 7.2], 150 mM NaCl, 5% [w/v] glycerol, 0.1% Igepal CA-630, 1 mM TCEP, and 1% BSA). Subsequently, 500 μl of purified $Ctf4_{CTD}$ protein at a concentration of 2 mg/ml was added to the Sepharose beads and binding was allowed to take place for an additional 1 hr at 4°C. The binding reaction was stopped by two consecutive washes with 1 ml of pull-down buffer and a final 1 ml wash with pull-down buffer without BSA. The Sepharose beads were mixed with SDS loading dye and $Ctf4_{CTD}$ interactions with the respective bait proteins were detected via SDS-PAGE. As a control, $Ctf4_{CTD}$ was tested for unspecific interaction with the Glutathione Sepharose resin and with GST and in both cases no interaction was detected.

Fluorescence Anisotropy

The lowest concentration of peptide at which the binding studies could be performed was determined via peptide calibration curves. Fluorescence anisotropy measurements were recorded in a PHERAstar Plus multi-detection plate reader (BMG Labtech) equipped with fluorescence polarization optic module (λ_{ex} = 485 nm; λ_{em} = 520 nm) at 25°C. Each data point is the mean of 200 flashes/well. The voltage gain was set by adjusting the target mP values of fluorescein-labeled peptides relative to that of fluorescein (35 mP). Serial dilutions of $Ctf4_{CTD}$ were made in 20 mM HEPES (pH 7.2), 140 mM KCl, and 5% (w/v) glycerol in the presence of 40 nM (Sld5 and Tof2) or 50 nM (Dna2) fluorescein-labeled peptide. For Dna2 peptide, each data point is the mean of three independent experiments and curve fitting to the experimental data was performed in pro Fit 6.2 (QuantumSoft) using a Levenberg-Marquardt fitting algorithm in combination with a Gaussian error distribution analysis. The interaction between $Ctf4_{CTD}$ and Tof2 peptide was too weak to be quantified reliably and data points were derived from a single measurement.

Pulse Field Gel Electrophoresis

A 30 ml aliquot of mid-exponential culture (about 2×10^8 cells) was taken for each sample and processed using the CHEF Yeast Genomic DNA Plug Kit (Bio-Rad, 170-3593), according to the manufacturer's instructions (6×10^8 cells per ml of agarose plug). A 3 mm slice of each plug was loaded on a 0.8% agarose (Certified Megabase Agarose, Bio-Rad, 161-3108) gel made in 1× Tris-Borate-EDTA buffer (TBE). Chromosomal DNA was separated using a CHEF-DR II system (Bio-Rad) with 1× TBE as running buffer, at 14°C for 90 hr at 3V/cm, with switch times ramping from 300 to 900 s. The gel was stained with 1 μg/ml ethidium bromide and photographed, before transfer of DNA to Hybond-XL membranes (GE Healthcare Life Sciences, RPN 203 S), using a VacuGene XL vacuum blotting system (GE Healthcare Life Sciences). Membranes were hybridized with a probe for the rDNA (Chromosome XII, 466875-467891), labeled with [α-32P]-dCTP using a Random Primed DNA labeling Kit (Roche, 11 004 760 001). For detection, membranes were exposed to BAS Imaging Plates (Fujifilm), which were then analyzed using a FLA-5100 scanner and AIDA Image Analysis software (Raytest).

ACCESSION NUMBERS

The accession numbers for the coordinates and structure factors reported in this paper are PDB: 5HOG (complex of $Ctf4_{CTD}$ with Dna2 207-223) and PDB: 5HOI ($Ctf4_{CTD}$ with Tof2 497-517).

SUPPLEMENTAL INFORMATION

Supplemental Information includes five figures and one table and can be found with this article online at http://dx.doi.org/10.1016/j.molcel.2016.06.009.

AUTHOR CONTRIBUTIONS

F.V. performed the experiments in Figures 1, 2B, 2D, 2E, 3A, 3C, 3F, 3G, 4A–4D, 5B–5F, 6, S1, S2A, S2C–S2I, and S5. A.C.S. and D.W. performed the experiments in Figures 2C, 3B, 3D, 3E, 4E, S3C, and S4B. M.A.O.B. established the conditions for CHEF gels. M.L.K. performed the fluorescence anisotropy assay in Figure S2B. D.M.-V. carried out the experiments in Figures S3B and S4A. K.L. and L.P. conceived the project and designed experiments in collaboration with F.V. and A.C.S. K.L. wrote the manuscript, with contributions and critical comments from the other authors.

ACKNOWLEDGMENTS

We gratefully acknowledge the support of the Medical Research Council (core grant MC_UU_12016/13), the Wellcome Trust (references 097945/B/11/Z for flow cytometry, 102943/Z/13/Z for award to K.L., and 104641/Z/14/Z for award to L.P.), and the Gates Cambridge PhD programme (A.C.S.) for funding our work. We thank Danesh Moazed for the rDNA silencing strains, Mark Peggie and Rachel Toth (Division of Signal Transduction Therapy in the MRC PPU) for construction of plasmid clones used in this project, and Frank Uhlmann for discussing unpublished data.

Received: January 19, 2016
Revised: May 12, 2016
Accepted: June 6, 2016
Published: July 7, 2016

REFERENCES

Araki, H., Hamatake, R.K., Johnston, L.H., and Sugino, A. (1991). DPB2, the gene encoding DNA polymerase II subunit B, is required for chromosome replication in Saccharomyces cerevisiae. Proc. Natl. Acad. Sci. USA *88*, 4601–4605.

Borges, V., Smith, D.J., Whitehouse, I., and Uhlmann, F. (2013). An Eco1-independent sister chromatid cohesion establishment pathway in S. cerevisiae. Chromosoma *122*, 121–134.

Buser, R., Kellner, V., Melnik, A., Wilson-Zbinden, C., Schellhaas, R., Kastner, L., Piwko, W., Dees, M., Picotti, P., Maric, M., et al. (2016). The replisome-coupled E3 ubiquitin ligase Rtt101Mms22 counteracts Mrc1 function to tolerate genotoxic stress. PLoS Genet. *12*, e1005843.

Cejka, P. (2015). DNA end resection: Nucleases team up with the right partners to initiate homologous recombination. J. Biol. Chem. *290*, 22931–22938.

Corbett, K.D., Yip, C.K., Ee, L.S., Walz, T., Amon, A., and Harrison, S.C. (2010). The monopolin complex crosslinks kinetochore components to regulate chromosome-microtubule attachments. Cell *142*, 556–567.

Drozdetskiy, A., Cole, C., Procter, J., and Barton, G.J. (2015). JPred4: a protein secondary structure prediction server. Nucleic Acids Res. *43* (W1), W389–394.

Gambus, A., van Deursen, F., Polychronopoulos, D., Foltman, M., Jones, R.C., Edmondson, R.D., Calzada, A., and Labib, K. (2009). A key role for Ctf4 in coupling the MCM2-7 helicase to DNA polymerase alpha within the eukaryotic replisome. EMBO J. *28*, 2992–3004.

Geil, C., Schwab, M., and Seufert, W. (2008). A nucleolus-localized activator of Cdc14 phosphatase supports rDNA segregation in yeast mitosis. Curr. Biol. *18*, 1001–1005.

Georgescu, R.E., Langston, L., Yao, N.Y., Yurieva, O., Zhang, D., Finkelstein, J., Agarwal, T., and O'Donnell, M.E. (2014). Mechanism of asymmetric polymerase assembly at the eukaryotic replication fork. Nat. Struct. Mol. Biol. *21*, 664–670.

Georgescu, R., Langston, L., and O'Donnell, M. (2015). A proposal: Evolution of PCNA's role as a marker of newly replicated DNA. DNA Repair (Amst.) *29*, 4–15.

Gosnell, J.A., and Christensen, T.W. (2011). Drosophila Ctf4 is essential for efficient DNA replication and normal cell cycle progression. BMC Mol. Biol. *12*, 13.

Hanna, J.S., Kroll, E.S., Lundblad, V., and Spencer, F.A. (2001). Saccharomyces cerevisiae CTF18 and CTF4 are required for sister chromatid cohesion. Mol. Cell. Biol. *21*, 3144–3158.

Hernández, H., and Robinson, C.V. (2007). Determining the stoichiometry and interactions of macromolecular assemblies from mass spectrometry. Nat. Protoc. *2*, 715–726.

Huang, J., Brito, I.L., Villén, J., Gygi, S.P., Amon, A., and Moazed, D. (2006). Inhibition of homologous recombination by a cohesin-associated clamp complex recruited to the rDNA recombination enhancer. Genes Dev. *20*, 2887–2901.

Im, J.S., Ki, S.H., Farina, A., Jung, D.S., Hurwitz, J., and Lee, J.K. (2009). Assembly of the Cdc45-Mcm2-7-GINS complex in human cells requires the Ctf4/And-1, RecQL4, and Mcm10 proteins. Proc. Natl. Acad. Sci. USA *106*, 15628–15632.

Johzuka, K., and Horiuchi, T. (2009). The cis element and factors required for condensin recruitment to chromosomes. Mol. Cell *34*, 26–35.

Kao, H.I., and Bambara, R.A. (2003). The protein components and mechanism of eukaryotic Okazaki fragment maturation. Crit. Rev. Biochem. Mol. Biol. *38*, 433–452.

Kim, S., Dallmann, H.G., McHenry, C.S., and Marians, K.J. (1996). Coupling of a replicative polymerase and helicase: a tau-DnaB interaction mediates rapid replication fork movement. Cell *84*, 643–650.

Kouprina, N., Kroll, E., Bannikov, V., Bliskovsky, V., Gizatullin, R., Kirillov, A., Shestopalov, B., Zakharyev, V., Hieter, P., Spencer, F., et al. (1992). CTF4 (CHL15) mutants exhibit defective DNA metabolism in the yeast Saccharomyces cerevisiae. Mol. Cell. Biol. *12*, 5736–5747.

Kunkel, T.A., and Burgers, P.M. (2014). Delivering nonidentical twins. Nat. Struct. Mol. Biol. *21*, 649–651.

Langston, L.D., Zhang, D., Yurieva, O., Georgescu, R.E., Finkelstein, J., Yao, N.Y., Indiani, C., and O'Donnell, M.E. (2014). CMG helicase and DNA polymerase ε form a functional 15-subunit holoenzyme for eukaryotic leading-strand DNA replication. Proc. Natl. Acad. Sci. USA *111*, 15390–15395.

Maculins, T., Nkosi, P.J., Nishikawa, H., and Labib, K. (2015). Tethering of SCF(Dia2) to the replisome promotes efficient ubiquitylation and disassembly of the CMG helicase. Curr. Biol. *25*, 2254–2259.

Mamnun, Y.M., Katayama, S., and Toda, T. (2006). Fission yeast Mcl1 interacts with SCF(Pof3) and is required for centromere formation. Biochem. Biophys. Res. Commun. *350*, 125–130.

Maric, M., Maculins, T., De Piccoli, G., and Labib, K. (2014). Cdc48 and a ubiquitin ligase drive disassembly of the CMG helicase at the end of DNA replication. Science *346*, 1253596.

Miles, J., and Formosa, T. (1992). Evidence that POB1, a Saccharomyces cerevisiae protein that binds to DNA polymerase alpha, acts in DNA metabolism in vivo. Mol. Cell. Biol. *12*, 5724–5735.

Mimura, S., Yamaguchi, T., Ishii, S., Noro, E., Katsura, T., Obuse, C., and Kamura, T. (2010). Cul8/Rtt101 forms a variety of protein complexes that regulate DNA damage response and transcriptional silencing. J. Biol. Chem. *285*, 9858–9867.

Park, H., and Sternglanz, R. (1999). Identification and characterization of the genes for two topoisomerase I-interacting proteins from Saccharomyces cerevisiae. Yeast *15*, 35–41.

Saka, K., Takahashi, A., Sasaki, M., and Kobayashi, T. (2016). More than 10% of yeast genes are related to genome stability and influence cellular senescence via rDNA maintenance. Nucleic Acids Res. *44*, 4211–4221.

Samora, C., Saksouk, J., Goswami, P., Wade, B.O., Singleton, M.R., Bates, P.A., Lengronne, A., Costa, A., and Uhlmann, F. (2016). Ctf4 links DNA replication with sister chromatid cohesion establishment by recruiting the Chl1 helicase to the replisome. Mol. Cell *63*, this issue, 371–384.

Sengupta, S., van Deursen, F., de Piccoli, G., and Labib, K. (2013). Dpb2 integrates the leading-strand DNA polymerase into the eukaryotic replisome. Curr. Biol. *23*, 543–552.

Simon, A.C., Zhou, J.C., Perera, R.L., van Deursen, F., Evrin, C., Ivanova, M.E., Kilkenny, M.L., Renault, L., Kjaer, S., Matak-Vinković, D., et al. (2014). A Ctf4 trimer couples the CMG helicase to DNA polymerase α in the eukaryotic replisome. Nature *510*, 293–297.

Tanaka, H., Katou, Y., Yagura, M., Saitoh, K., Itoh, T., Araki, H., Bando, M., and Shirahige, K. (2009). Ctf4 coordinates the progression of helicase and DNA polymerase alpha. Genes Cells *14*, 807–820.

van Deursen, F., Sengupta, S., De Piccoli, G., Sanchez-Diaz, A., and Labib, K. (2012). Mcm10 associates with the loaded DNA helicase at replication origins and defines a novel step in its activation. EMBO J. *31*, 2195–2206.

Warbrick, E. (1998). PCNA binding through a conserved motif. BioEssays *20*, 195–199.

Williams, D.R., and McIntosh, J.R. (2002). mcl1+, the Schizosaccharomyces pombe homologue of CTF4, is important for chromosome replication, cohesion, and segregation. Eukaryot. Cell *1*, 758–773.

Yao, N.Y., and O'Donnell, M. (2010). SnapShot: the replisome. Cell *141*, 1088, 1088 e1081.

Yeeles, J.T., Deegan, T.D., Janska, A., Early, A., and Diffley, J.F. (2015). Regulated eukaryotic DNA replication origin firing with purified proteins. Nature *519*, 431–435.

Zhu, W., Ukomadu, C., Jha, S., Senga, T., Dhar, S.K., Wohlschlegel, J.A., Nutt, L.K., Kornbluth, S., and Dutta, A. (2007). Mcm10 and And-1/CTF4 recruit DNA polymerase alpha to chromatin for initiation of DNA replication. Genes Dev. *21*, 2288–2299.

Molecular Cell, Volume *63*

Supplemental Information

Ctf4 Is a Hub in the Eukaryotic Replisome that Links Multiple CIP-Box Proteins to the CMG Helicase

Fabrizio Villa, Aline C. Simon, Maria Angeles Ortiz Bazan, Mairi L. Kilkenny, David Wirthensohn, Mel Wightman, Dijana Matak-Vinkovíc, Luca Pellegrini, and Karim Labib

Villa et al Supplementary Figure 1

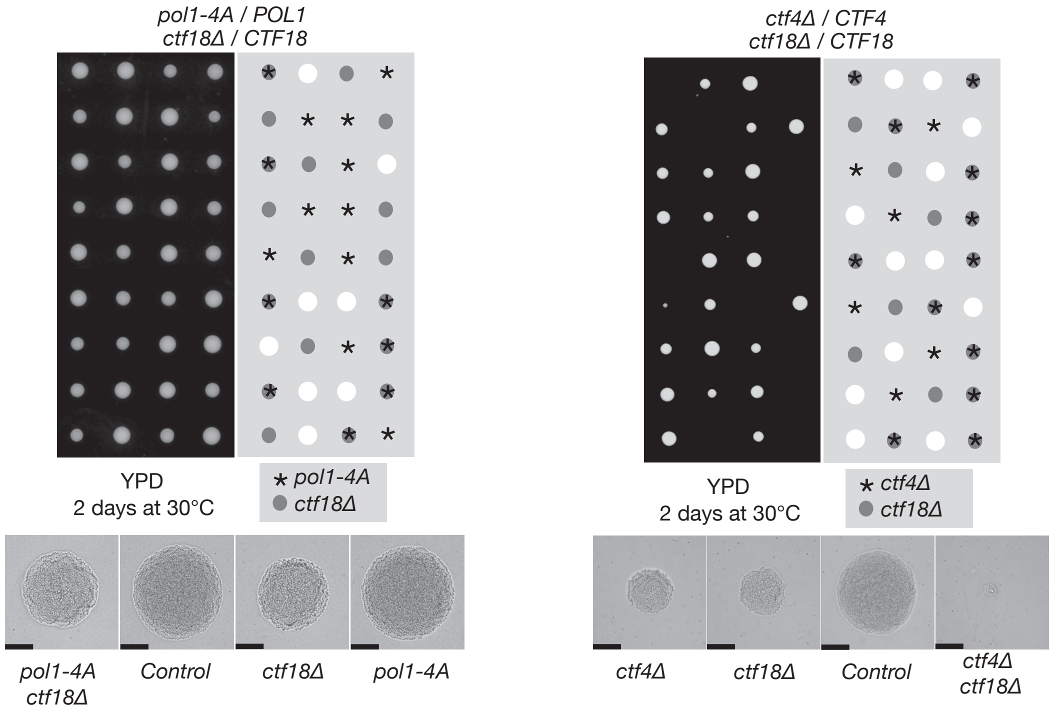

Villa et al Supplementary Figure 2

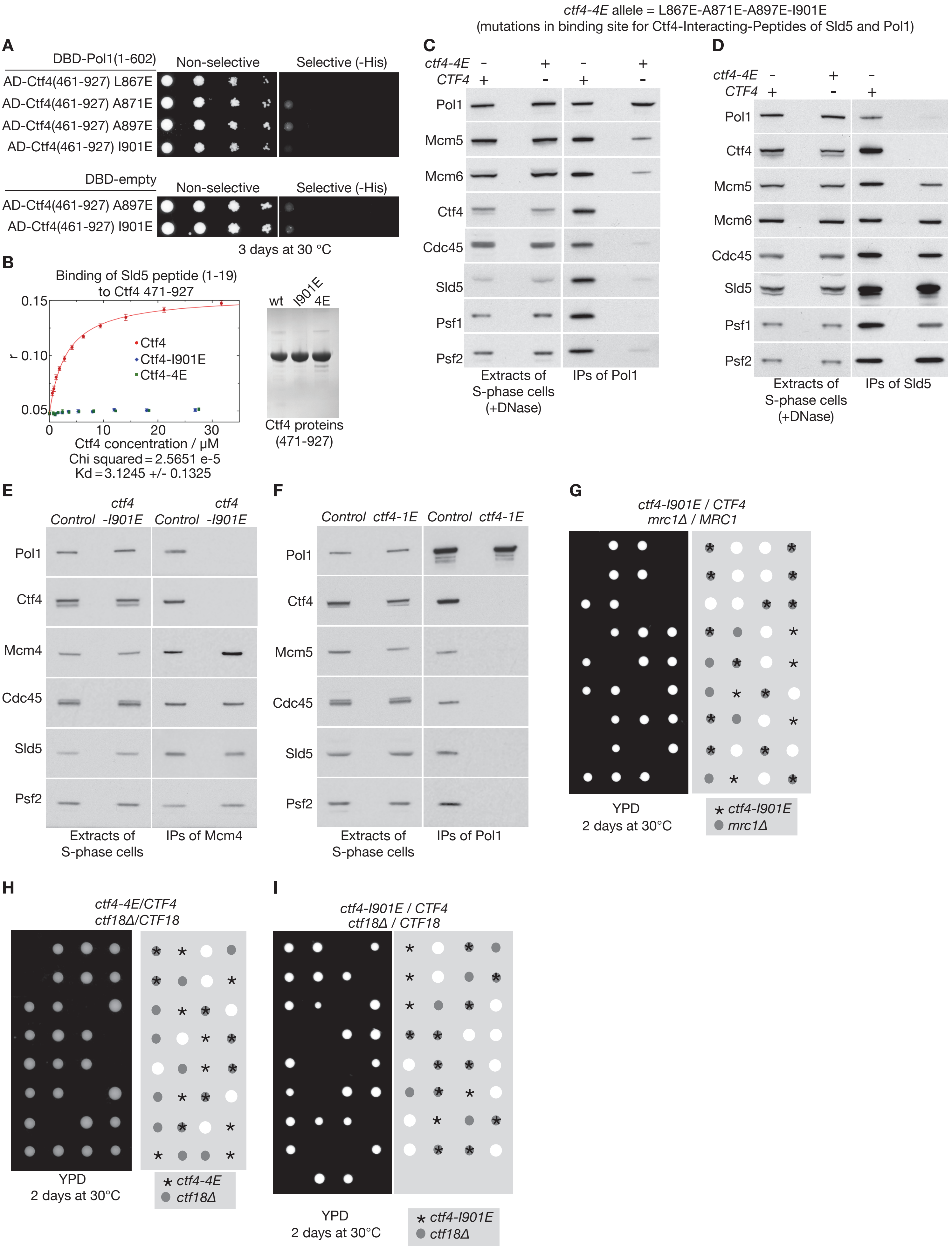

Villa et al Supplementary Figure 3

A

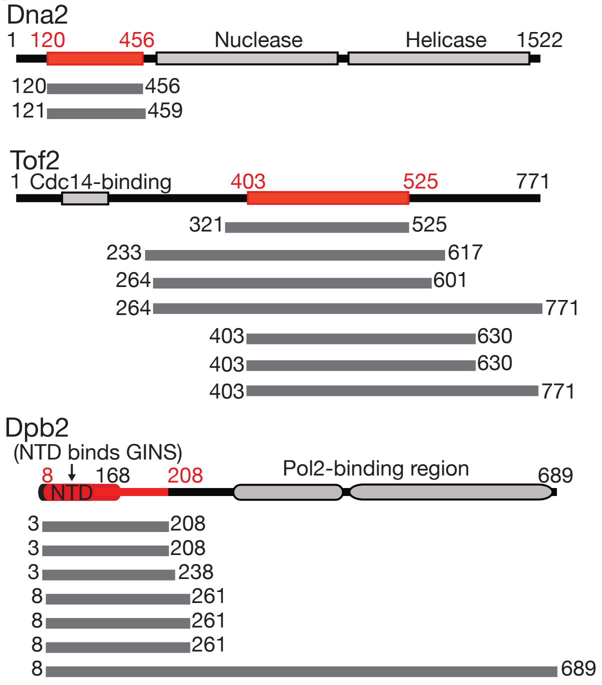

B

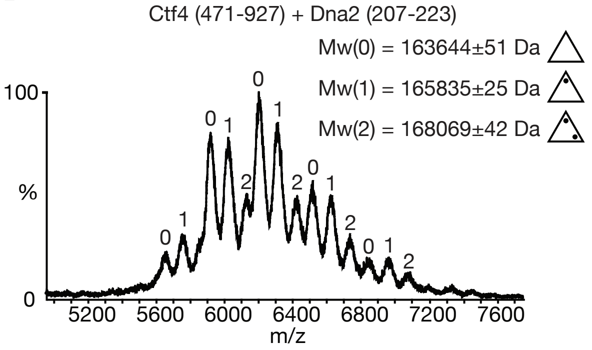

C

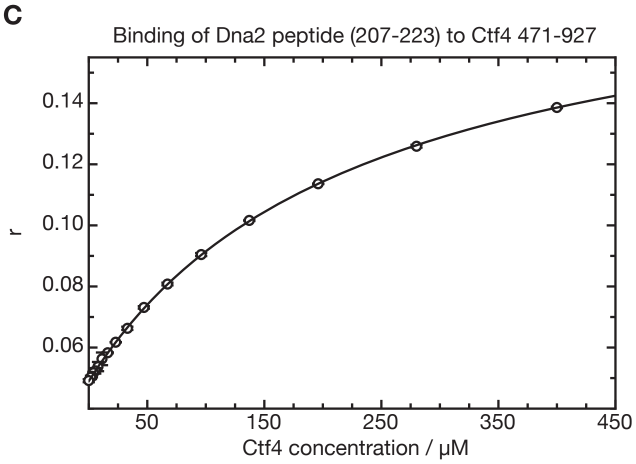

Chi squared = 5.1970
Goodness of fit = 0.9903

Parameters: Kd = 229.0088
Standard deviations: Kd = 12.7404

Kd 95% Confidence intervals 205.6937 ... 256.0578
(based on 500 converging iterations)

D

14 Budding yeast proteins with peptides matching the pattern:
[ND]-X-D-D-I-L-X-X-J,
where [ND] = N or D, J = hydrophobic residue and
X = any residue

Sld5 4-NIDDILAEL-12
(subunit of GINS component of CMG DNA helicase)

Dna2 210-NIDDILDDI-218
(Nuclease with role in DNA replication and recombination)

Pol1 139-NFDDILGEF-147
(Catalytic subunit of DNA polymerase alpha)

Chl1 701-DGDDILSGY-709
(DNA helicase important for establishment of sister chromatid cohesion)

Lcd1/Ddc2 13-DDDDILLEL-21
(essential partner of Mec1 protein kinase for S-phase checkpoint response)

Isw2 681-DIDDILKKG-689
(ATPase involved in chromatin remodeling)

Irc20 734-NIDDILISM-742
(E3 ubiquitin ligase and putative helicase involved in recombination)

Rfx1 681-NLDDILEMM-689
(Transcriptional repressor of DNA-damage induced genes)

Pex19 176-NIDDILSQL-184
(chaperone and import receptor for peroxisomal membrane proteins)

Cog7 9-DDDDILSMF-17
(Protein trafficking in the Golgi)

Pdr8 204-NQDKDDILSLL-212
(Transcription factor controlling membrane transporters)

Vps45 453-DKDDILTEL-461
(Vacuolar Protein Sorting)

Tub4 115-NQDDILNKI-123
(Gamma Tubulin)

Sia1 590-NLDDILDEI-598
(Unknown function)

Villa et al Supplementary Figure 4

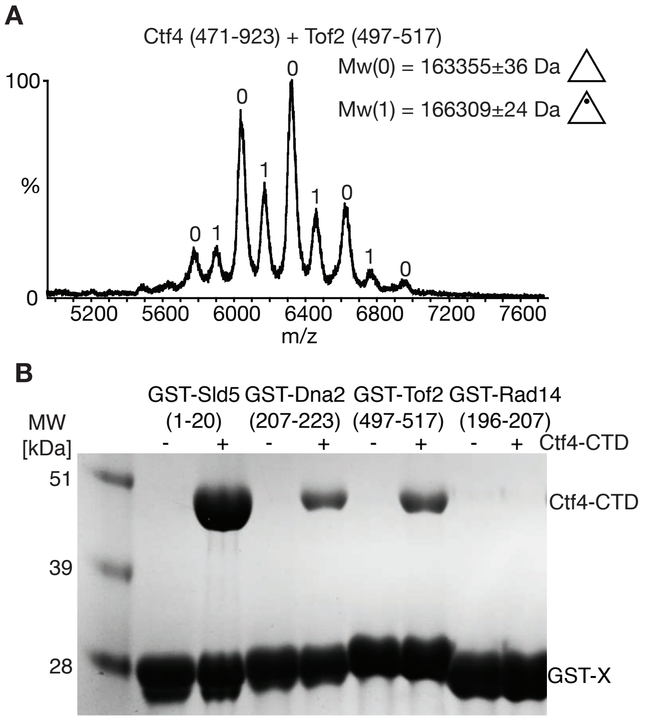

Villa et al Supplementary Figure 5

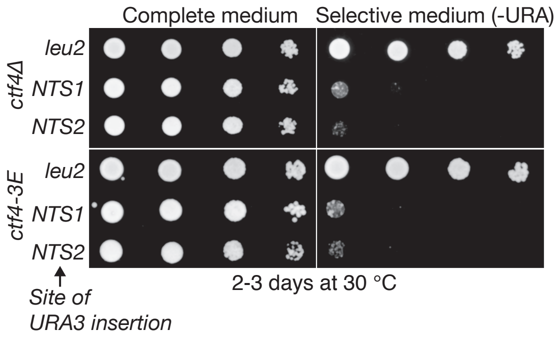

Legends to Supplemental Figures and Table

Figure S1, related to main Figure 1

pol1-4A does not share the synthetic lethal phenotype of *ctf4Δ* with *ctf18Δ*. The indicated diploids were sporulated and tetrads were dissected. The photos in the lower panels were taken after 20 hours growth at 30°C (scale bars = 50μm), and genotypes were determined by replica plating after two days of growth.

Figure S2, related to main Figure 2

Additional phenotypes of mutations in the C-terminal peptide-binding domain of Ctf4. (**A**) Single mutations in the C-terminal peptide-binding domain of Ctf4 block interaction with Pol1 amino terminus in the yeast two-hybrid assay. (**B**) Fluorescence anisotropy showing that the Ctf4-I901E and Ctf4-4E mutations blocked binding to a peptide containing the Sld5 CIP-box. (**C**) Extracts of *ctf4-4E POL1-6HA* (YFV12) and *POL1-6HA* control cells (YMP262-1) were grown as in Figure 2D. Pol1-6HA was isolated from cell extracts by immunoprecipitation on anti-HA beads, and the associated proteins monitored by immunoblotting. (**D**) A similar experiment was performed with *ctf4-4E TAP-SLD5* (YFV9) and *TAP-SLD5* control cells (YSS47). (**E**) Extracts of *ctf4-I901E MCM4-9MYC* (YFV33) and *MCM4-9MYC* control cells (YSS75) were grown as in Figure 2D. Mcm4-9MYC was isolated from cell extracts by immunoprecipitation on anti-MYC beads, and the associated proteins monitored by immunoblotting. (**F**) A similar experiment was performed with *ctf4-I901E POL1-6HA* (YFV32) and *POL1-6HA* control

cells (YMP262-1). (**G**) *ctf4-I901E* is synthetic lethal with *mrc1Δ*. Cells were processed as in Figure 1A. (**H**) *ctf4-4E* is synthetic lethal with *ctf18Δ*. (**I**) *ctf4-I901E* is synthetic lethal with *ctf18Δ*.

Figure S3, related to main Figure 3

A screen for novel partners of Ctf4 461-927, and characterisation of the CIP-box of Dna2. (**A**) Summary of the novel hits in the two hybrid screen against Ctf4 461-927. Each grey line indicates a single hit in the screen, and the red segments indicate the minimal fragment of each protein that is predicted to interact with Ctf4. (**B**) Native mass spectrometry was used to show that Ctf4 471-927 binds directly to the Dna2 CIP *in vitro*. (**C**) Fluorescence anisotropy confirmed binding of Ctf4 471-927 with a Kd of 229μM. (**D**) Results of Pattern Match search of the yeast proteome (http://www.yeastgenome.org/cgi-bin/PATMATCH/nph-patmatch), using the indicated sequence that is based on the CIP-boxes of Sld5-Pol1-Dna2.

Figure S4, related to main Figure 4

The Tof2 CIP binds directly to Ctf4 471-927 *in vitro*. (**A**) Native mass spectrometry showed that Ctf4 471-927 binds directly to the Tof2 CIP-box. (**B**) GST-fusions of the indicated peptides were tested for binding to Ctf4 471-927. A peptide from the Rad14 protein provided a negative control.

Figure S5, related to main Figure 6

Transcriptional silencing in the rDNA repeats is not defective in *ctf4-3E* or *ctf4Δ* cells. Cells were processed as in Figure 6D.

Table S1, related to main figure 1

Strains used in this study - all based on the W303 yeast genetic background, except for the yeast two-hybrid strain PJ69-4A.

Strain	Genotype
W303-1	*MAT**a** ade2-1 ura3-1 his3-11,15 trp1-1 leu2-3,112 can1-100 / MATα ade2-1 ura3-1 his3-11,15 trp1-1 leu2-3,112 can1-100*
W303-1a	*MAT**a** ade2-1 ura3-1 his3-1 trp1-1 leu2-3, 112 can1-100*
PJ69-4A	*MAT**a** trp1-901 leu2-3,112 ura3-52 his3-200 gal4Δ gal80Δ LYS2::GAL1-HIS3 GAL2-ADE2 met2::GAL7-la*
YAG132	*MAT**a** / MATα CTF4 / ctf4Δ::kanMX MRC1 / mrc1Δ::hphNT*
YCE449	*MAT**a** pol1-4A (URA3)*
YDP14	*MAT**a** / MATα CTF4 / ctf4Δ::kanMX CTF18 / ctf18Δ::hphNT*
YFV9	*MAT**a** ctf4-4E TAP-SLD5 (kanMX) pep4Δ::ADE2*
YFV11	*MAT**a** ctf4-4E*
YFV12	*MAT**a** ctf4-4E POL1-6HA (K.l. TRP1) pep4Δ::ADE2*
YFV13	*MAT**a** ctf4-4E MCM4-9MYC (kanMX) pep4Δ::ADE2*
YFV17	*MAT**a** dna2-4A-9MYC* (*K.l.TRP1*)
YFV21	*MAT**a** GAL-DNA2-9MYC* (*kanMX & K.l. TRP1*) *pep4Δ::ADE2*
YFV22	*MAT**a** GAL-DNA2-9MYC* (*kanMX & K.l. TRP1*) TAP-CTF4 (*kanMX*) *pep4Δ::ADE2*
YFV30	*MAT**a*** tof2*Δ::URA3*
YFV31	*MAT**a** ctf4-3E*
YFV33	*MAT**a** ctf4-I901E MCM4-9MYC (kanMX) pep4Δ::ADE2*
YFV36	*MAT**a** tof2-4A*
YFV47	*MAT**a** ura3:: pRS306-GAL-ProteinA-CBP-TEV-TOF2 (URA3) pep4Δ::ADE2*
YFV55	*MAT**a*** / *MATα ctf4-4E MRC1 / mrc1Δ::K.l.TRP1*
YFV56	*MAT**a*** / *MATα ctf4-4E CTF18 / ctf18Δ::K.l.TRP1*
YFV57	*MAT**a*** / *MATα ctf4-I901E CTF18 / ctf18Δ::K.l.TRP1*
YFV58	*MAT**a*** / *MATα ctf4-I901E MRC1 / mrc1Δ::K.l.TRP1*
YFV59	*MAT**a** / MATα MRC1 / mrc1Δ::K.l.TRP1 POL1 / pol1-4A (URA3)*
YFV60	*MAT**a** / MATα CTF18 / ctf18Δ::K.l. TRP1 POL1 / pol1-4A (URA3)*
YFV62	*MAT**a*** / *MATα DNA2 / dna2-4A MEC1 / mec1Δ::HIS sml1Δ::ADE2/ sml1Δ::ADE2*
YHM160	*MAT**a** ctf4Δ::kanMX*
YMP262-1	*MAT**a** POL1-6HA (K.l. TRP1) pep4Δ::ADE2*
YSS47	*MAT**a** TAP-SLD5 (kanMX) pep4Δ::ADE2*
YSS75	*MAT**a** MCM4-9MYC (kanMX) pep4Δ::ADE2*
